# The Proteome of Exosomes at Birth Predicts Insulin Resistance, Adrenarche and Liver Fat in Childhood

**DOI:** 10.3390/ijms26041721

**Published:** 2025-02-18

**Authors:** Marta Díaz, Tania Quesada-López, Francesc Villarroya, Paula Casano, Abel López-Bermejo, Francis de Zegher, Lourdes Ibáñez

**Affiliations:** 1 Endocrinology Department, Institut de Recerca Sant Joan de Déu, University of Barcelona, 08950 Barcelona, Spain; paula.casano@sjd.es; 2 Centro de Investigación Biomédica en Red de Diabetes y Enfermedades Metabólicas Asociadas (CIBERDEM), Instituto de Salud Carlos III, 28029 Madrid, Spain; 3 Department of Biomedicine, Institut de Recerca Hospital de la Santa Creu i Sant Pau, 08041 Barcelona, Spain; tquesada@gmail.com; 4 Network Biomedical Research Center of Physiopathology of Obesity and Nutrition (CIBEROBN), Health Institute Carlos III, 28029 Madrid, Spain; fvgombau@gmail.com; 5 Biochemistry and Molecular Biomedicine Department, Institute of Biomedicine, University of Barcelona, 08007 Barcelona, Spain; 6 Institut de Recerca Sant Joan de Déu, Esplugues, 08950 Barcelona, Spain; 7 Pediatric Endocrinology Research Group, Girona Institute for Biomedical Research (IDIBGI), Faculty of Medicine, University of Girona and Dr. Josep Trueta Hospital, 17007 Girona, Spain; alopezbermejo@idibgi.og; 8 Leuven Research & Development, University of Leuven, 3000 Leuven, Belgium; francis.dezegher@kuleuven.be

**Keywords:** proteome, exosomes, SGA, liver fat, lipid metabolism, insulin signaling

## Abstract

It is unknown whether there are differentially expressed proteins (DEPs) in the circulating exosomes of appropriate- vs. small-for-gestational-age (AGA vs. SGA) infants, and if so, whether such DEPs relate to measures of endocrine–metabolic health and body composition in childhood. Proteomic analysis in cord-blood-derived exosomes was performed by label-free quantitative mass spectrometry in AGA (n = 20) and SGA infants (n = 20) and 91 DEPs were identified. Enrichment analysis revealed that they were related to complement and coagulation cascades, lipid metabolism, neural development, PI3K/Akt and RAS/RAF/MAPK signaling pathways, phagocytosis and focal adhesion. Protein–protein interaction (PPI) analysis identified 39 DEPs involved in the pathways enriched by the KEGG and Reactome. Those DEPs were associated with measures of adiposity and insulin resistance and with liver fat at age 7 (all *p* < 0.01). Multivariate linear regression analysis uncovered that two DEPs (up-regulated in SGA), namely PCYOX1 (related to adipogenesis) and HSP90AA1 (related to lipid metabolism and metabolic-dysfunction-associated steatotic liver disease progression), were independent predictors of the hepatic fat fraction at age 7 (β = 0.634; *p* = 0.002; R^2^ = 52% and β = 0.436; *p* = 0.009; R^2^ = 24%, respectively). These data suggest that DEPs at birth may predict insulin resistance, adrenarche and/or ectopic adiposity in SGA children at age 7, when an early insulin-sensitizing intervention could be considered.

## 1. Introduction

Most tissues secrete exosomes, which are extracellular vesicles that may influence the function of distant cells. Exosome cargo (i.e., proteins, lipids, mRNAs, non-coding RNAs, DNA) and its release reflect the cell origin, vary with physiological and/or pathological states and can alter biological pathways [1]. Exosomes play a critical role in pregnancy by regulating processes such as the mother’s immunological and metabolic adaptations; as gestation advances, placental cells of maternal or embryonic origin raise the number of circulating exosomes, especially in the context of preeclampsia, gestational diabetes, intrauterine growth restriction and/or preterm delivery [2]. Distinct exosomal proteome patterns are emerging as biomarkers of cancer [3], diabetes [4], obesity [5], neurodegenerative disorders [6] and gestational complications such as preeclampsia [7], gestational diabetes [8] and fetal death [9].

The sequence of less prenatal weight gain and more postnatal weight gain confers a risk for ectopic (hepato-visceral) fat accumulation, insulin resistance (IR), polycystic ovary syndrome (PCOS), type 2 diabetes (T2D) and cardiovascular disease [10,11,12,13]. However, the mechanisms underlying these associations remain to be fully delineated.

At birth, the circulating exosomes of small-for-gestational-age infants [SGA, birthweight (BW) and/or length ≤−2.0 SD] [14] are larger in size than those of appropriate-for-GA (AGA) infants and, after spontaneous catch-up growth at ages 2 yr and 7 yr, they are lower in number [15].

In the present study, we assessed whether there are differentially expressed proteins (DEPs) in cord-blood-derived exosomes of AGA vs. SGA-catch-up infants at birth and, if so, whether such DEPs relate to measures of endocrine–metabolic health and body composition in childhood.

## 2. Results

### 2.1. Longitudinal Auxological, Endocrine–Metabolic and Imaging Variables

Table S1 depicts selected parameters of the study population by BW subgroup. As previously reported, SGA infants displayed at birth lower circulating levels of insulin-like growth factor-1 (IGF-1) and high-molecular-weight-adiponectin (HMW-adip), less total and abdominal fat and lean mass and a lower bone mineral content and density as compared to AGA infants [15,16,17]. At age 2 yr, all SGA infants had completed their catch-up and had normalized their circulating IGF-1 levels, as well as their fat and lean mass. By age 7 yr, SGA children displayed higher levels of IGF-1 and dehydroepiandrosterone-sulfate (DHEAS, a marker of adrenarche), were more insulin resistant [as judged by the homeostasis model assessment for IR (HOMA-IR)] and exhibited a higher amount of liver fat [by magnetic resonance imaging (MRI)].

### 2.2. Exosome Characterization

Exosomes were characterized for their number and size distribution by Nanoparticle Tracking Analysis (NTA), as previously reported [15]. The mean size of exosomes isolated from cord blood was within the range of 139–143 nm (Figure S1).

### 2.3. Label-Free Quantitative Proteomics

**Figure 1:**
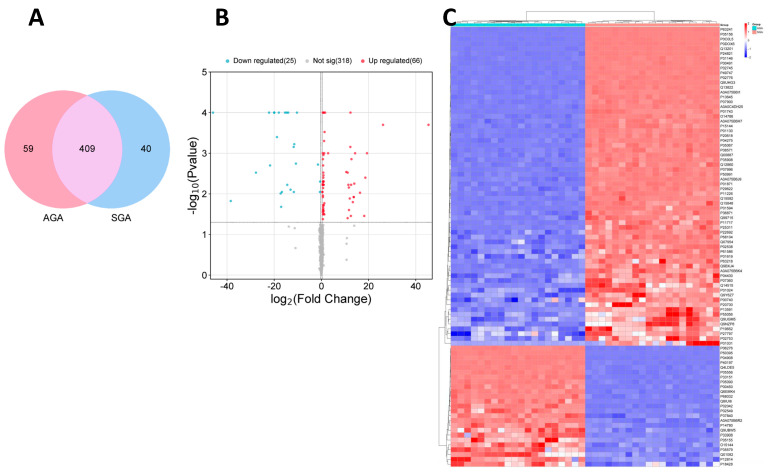
Proteomic analysis in cord-blood-derived exosomes from appropriate- (AGA, n = 20) and small-for-gestational-age (SGA, n = 20) infants (**A**) Venn diagram depicting the proteins identified in cord-blood-derived exosomes from AGA (n = 20) and SGA (n = 20) infants. (**B**) Volcano plot depicting the significance of the data differences from AGA (n = 20) vs. SGA (n = 20) infants. The X-axis represents the protein difference (log2-transformed fold changes), and the Y-axis represents the corresponding -log10-transformed P values. Red dots and blue dots indicate significantly up-regulated and down-regulated proteins, respectively, in the SGA subgroup. Gray dots symbolize proteins with no significant change. (**C**) Hierarchical clustering of differentially expressed proteins. Columns represent different samples according to subgroups (n = 20 AGA, left panel; n = 20 SGA, right panel), and rows represent different proteins, clustered by log10 (protein expression value + 1) values. Data in each row are normalized; red indicates a high-expression protein, blue indicates a low-expression protein.

N = 508 proteins were identified in cord-blood-derived exosomes by label-free quantitative proteomics; of those, n = 409 were detected in both AGA and SGA subgroups; n = 59 exclusively in the AGA subgroup; and n = 40 only in the SGA subgroup (Figure 1A). The proteins exclusively identified in the AGA or SGA subgroups are shown in Tables S2A and S2B, respectively.

N = 91 (out of 409) DEPs were found between the AGA and SGA subgroups; of those, n = 66 were up-regulated and n = 25 down-regulated in the SGA subgroup (Table S3; Figure 1B).

The correlation heatmap clustered separated samples from the two subgroups, indicating a significant difference in exosomal proteomes between the AGA and SGA subpopulations (Figure 1C).

**Figure 2:**
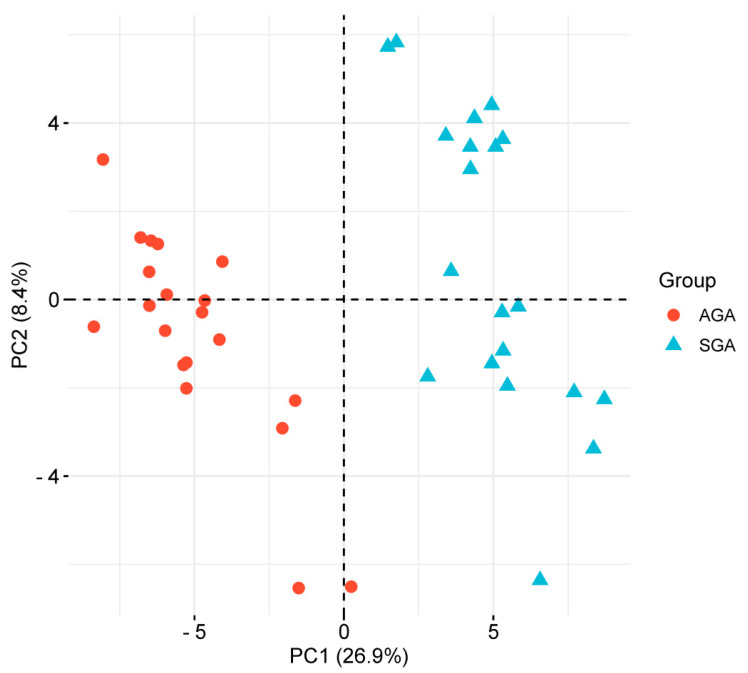
Principal component analysis (PCA) of the proteomic profile of cord-blood-derived exosomes from appropriate-for-gestational age (AGA, n = 20, red dots) or small-for-gestational age (SGA, n = 20, blue triangles) infants. The PCA score plots show that samples from AGA and SGA infants were clustered separately. The first two principal components (PC1 and PC2) explained 35.3% of the variance between subgroups.

The principal component analysis (PCA) also strengthened the distinct proteome pattern in SGA children, discerning a spatial separation from the AGA subgroup (Figure 2). The two principal components explained 35.3% of the variance observed between study subgroups.

Proteins with no significant expression changes (n = 318 out of n = 409) between subgroups are shown in Table S4.

### 2.4. Functional Enrichment Analysis of Differentially Expressed Proteins (DEPs)

**Figure 3:**
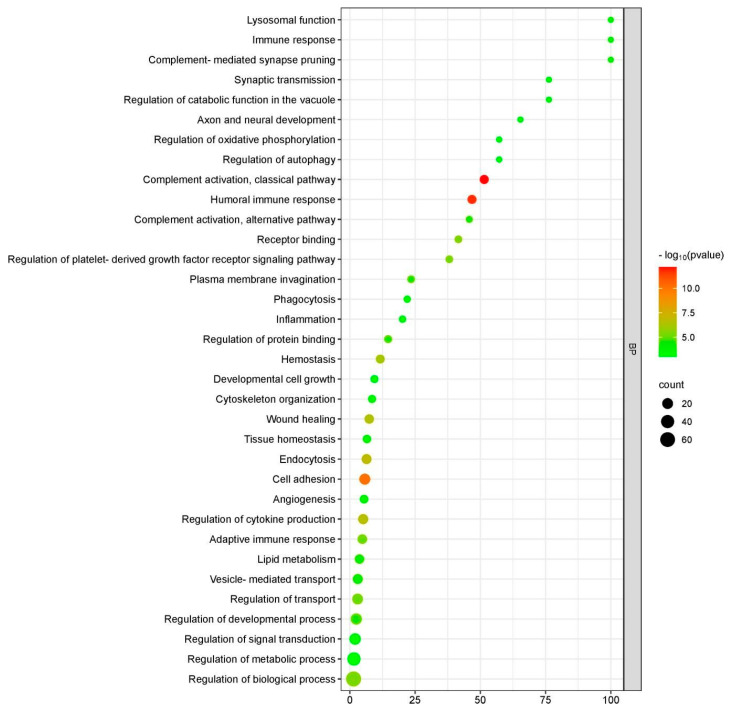
GO singular enrichment analysis (“biological process” category) using Fisher’s exact test. Dots represent term enrichment; *p*-values indicating the statistical significance of the enrichment are represented along a gradient color from light green (less significant) to red (most significant). The sizes of the dots represent the count of differentially expressed genes belonging to each term; y-axis: enriched GO term; x-axis: gene ratio [#significant genes/#annotated genes (%)].

Gene ontology (GO) enrichment analysis was performed to reveal the significantly enriched biological functions of the DEPs; Figure 3 depicts the statistically significant items in biological processes (BPs). These proteins were mainly related to immune response, complement activation, cell adhesion and regulation of metabolic processes. The DEPs in cellular components (CCs) were mainly enriched in cell adhesion, the cytoskeleton and cell organelles. Enriched molecular functions were mainly associated with binding, catalytic activity and structural components of the extracellular matrix and cytoskeleton.

The KEGG analysis revealed that these proteins play a role in complement and coagulation processes, cell adhesion, cytoskeletal dynamics, lipid metabolism and cell growth and survival (Table 1).

In addition, enrichment analysis was also performed in those proteins identified only in the AGA or SGA subgroups (Tables S5A and S5B, respectively).

**Figure 4:**
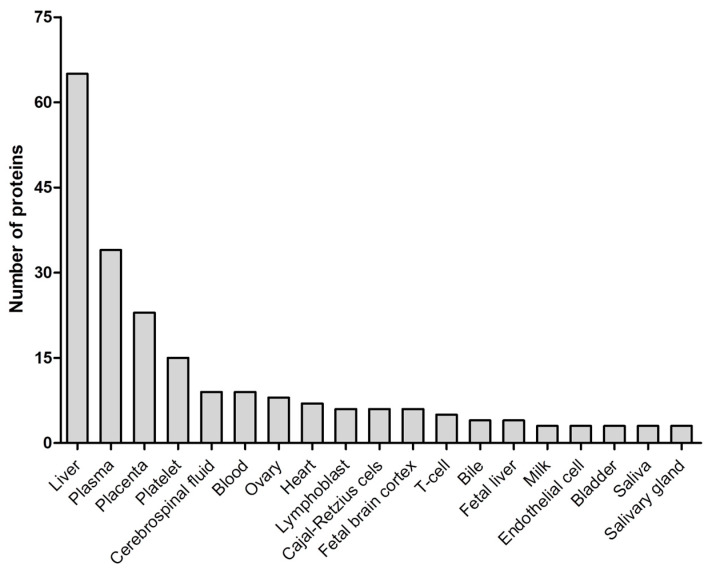
Tissue origins of the differentially expressed proteins in cord blood exosomes according to the DAVID knowledgebase.

Tissue expression mapping according to the Database for Annotation Visualization, and Integrated Discovery (DAVID) knowledgebase evidenced a high enrichment of liver-derived proteins (n = 65, Figure 4).

### 2.5. Network Analysis of Differentially Expressed Proteins (DEPs)

**Figure 5:**
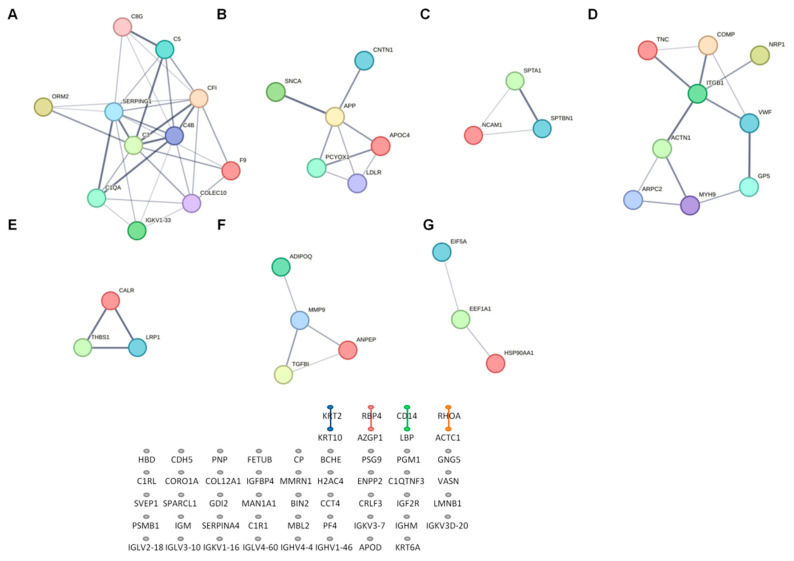
Network analysis of differentially expressed proteins in appropriate-for-gestational-age (AGA, n = 20) vs. small-for-gestational-age (SGA, n = 20) infants. Functional annotation analysis of subnetworks revealed clusters of proteins involved in (**A**) complement activation and coagulation cascades; (**B**) lipid metabolism and neural development; (**C**) the RAS/RAF/MAP kinase cascade; (**D**) focal adhesion and the PI3K/Akt signaling pathway; (**E**) phagocytosis; (**F**) adipocyte-derived extracellular vesicles; and (**G**) Heat shock factor 1 (HSF1) activation.

The protein–protein interaction (PPI) network was generated using STRING. The complete network was composed of 77 nodes and 243 edges, with an average node degree of 6.31 and PPI enrichment *p*-value of <1.0 × 10^−16^. Cluster analysis of the network showed the presence of seven subnetworks according to the total interaction strength (Figure 5). Those proteins were involved in the complement and coagulation system (*p* = 1.0 × 10^−16^), in the focal adhesion and phosphatidylinositol 3′-kinase (PI3K)/protein Kinase B (Akt) signaling pathway (*p* = 8.4 × 10^−9^), in lipid metabolism and neural development (*p* = 6.8 × 10^−8^), in the Ras–mitogen-activated protein RAS/RAF/MAP kinase cascade (*p* = 1.2 × 10^−4^), in the regulation of phagocytosis (*p* = 1.1 × 10^−3^) and in heat shock factor 1 (HSF1) activation (*p* = 2.09 × 10^−2^). Interestingly, several proteins enriched in adipocyte-derived extracellular vesicles were also found to form a network (*p* = 8.0 × 10^−4^).

### 2.6. Receiver Operating Characteristics (ROC) Analysis

To assess the discriminatory capacity of the DEPs belonging to the different subnetworks, a receiver operating characteristics (ROC) analysis was conducted. The top 10 up-regulated proteins and top 9 down-regulated proteins were able to differentiate the two subgroups according to BW with a high degree of confidence (AUC ≥ 0.8) (Figures S2A and S2B, respectively).

### 2.7. Correlation Analysis

A correlation matrix was formed, including the top 19 dysregulated proteins and selected variables at birth (Figure S3), at age 2 yr (Figure S4) and at age 7 yr (Figure S5).

At birth, up-regulated proteins (ORM2, F9, COLEC10, APOC4, LDLR, PCYOX1, CALR, NCAM1, EEF1A1, HSP90AA1) were inversely associated with anthropometric variables, with circulating IGF-1 and HMW-adip concentrations and with body fat; in addition, LDLR, PCYOX1 and CALR showed a negative correlation with HOMA-IR.

At age 2 yr, F9, PCYOX1 and LDLR were positively correlated with the Z-score change from BW to body mass index (BMI). PCYOX1 expression levels were negatively associated with HMW-adip and positively with HOMA-IR along with HSP90AA1.

At age 7 yr, all selected proteins were positively correlated with the Z-score change from BW to BMI and negatively correlated with HMW-adip. NCAM1 and EEF1A1 were correlated positively with DHEAS (r = 0.623, *p* = 0.010 and r = 0.616, *p* = 0.011, respectively). Liver fat was positively associated with Prenylcysteine Oxidase 1 (PCYOX1, involved in the regulation of adipogenesis) and Heat shock protein 90 Alpha Family Class A Member 1 [HSP90AA1 involved in lipid metabolism and metabolic-dysfunction-associated steatotic liver disease (MASLD) progression].

Down-regulated proteins (SERPING1, SNCA, ACTN1, ARPC2, MYH9, SPTA1, SPTBN1, MMP9, GP5) were positively correlated with anthropometric variables and with total and abdominal fat at birth.

At age 2 yr, SERPING1 and MYH9 were negatively correlated with the Z-score change from BW to BMI and with HOMA-IR.

At age 7 yr, all proteins were negatively correlated with the Z-score change from BW to BMI. ARPC2, MYH9 and SPTA1 were inversely correlated with HOMA-IR, whereas ARPC2, SPTB1 and MMP9 were positively correlated with HMW-adip. ACTN1, ARPC2 and MYH9 were associated negatively with DHEAS (r = −0.601, *p* = 0.018; r = −0.689, *p* = 0.009 and r = −0.781, *p* = 0.001, respectively). The expression levels of SERPING1, SNCA and MYH9 were negatively correlated with liver fat.

Multivariate linear regression analysis showed that the expression levels of PCYOX1 and HSP90AA1 were independent predictors of the percentage of liver fat at age 7 yr (β= 0.432; *p* = 0.007; R^2^ = 33% and β= 0.689; *p* = 0.005; R^2^ = 38%, respectively) (Table 2).

Moreover, COLEC10 and CALR were independent predictors of fat mass and abdominal fat at age 2 years (β = 0.811; *p* = 0.035; R^2^ = 61% and β = 0.738; *p* = 0.037; R^2^ = 47%, respectively). The Z-score change from BW to BMI at age 7 yr was predicted by SPTBN1 (β = −0.702; *p* = 0.035; R^2^ = 42%), whilst circulating HMW-adip levels at age 7 yr were predicted by MMP9 (β = −0.939; *p* = 0.010; R^2^ = 84%) (Table S6).

## 3. Discussion

In the present study, we report that AGA and SGA infants have distinct proteomic profiles in cord-blood-derived exosomes, and we reveal associations between DEPs and markers of neonatal body composition and early weight gain, as well as with markers of adrenarche, IR and liver fat at age 7 yr.

We detected n = 91 DEPs in SGA vs. AGA infants (66 up-regulated and 25 down-regulated). PPI analysis identified 39 DEPs belonging to subnetworks mainly involved in complement and coagulation cascades, lipid metabolism, neural development, PI3K/Akt and RAS/RAF/MAPK signaling pathways, phagocytosis, focal adhesion and HSF1 activation.

The complement system plays an essential role in the innate immune response [18]. Complement over-activation due to loss-of-function mutations in regulatory proteins predisposes individuals to adverse pregnancy outcomes [19]. For example, mice deficient in C1q experience abnormal placentation and fetal loss [20], whereas C3-deficient mice deliver growth-restricted fetuses [21]. In addition, connections between complement and whole-body metabolism have been reported. In this regard, perturbations in serum complement activation beyond the homeostatic level have been related to obesity, IR and T2D [22,23]. Here, we found that the expression levels of proteins involved in complement activation were associated with the Z-score change from BW to BMI at age 7 yr and with HOMA-IR, suggesting a role of these proteins in the development of excess adiposity in children with an upward mismatch between prenatal weight gain, as judged by BW, and postnatal weight gain, as judged by childhood BMI [24]. These results align well with a previous report unveiling the expression of complement proteins in human intra-abdominal adipose tissue, linking adipose tissue inflammation, obesity and IR [25]. Furthermore, adipose tissue expansion is mediated by extracellular matrix (ECM) remodeling [26]. Proteomic analysis revealed an altered expression in focal adhesion proteins needed for the cell-ECM linkage in SGA infants. Thus, it is tempting to speculate that disturbances in ECM–complement signaling could account, at least in part, for the limited capacity of white adipose tissue expansion in SGA-catch-up children, leading to ectopic fat accumulation. This hypothesis is supported by the recent identification of the integrin leukocyte adhesion molecule [leukocyte function-associated antigen-1 (LFA-1)] connecting integrins, the complement and metabolism in a new triangular functional relationship [27].

We uncovered changes in the expression pattern of proteins involved in insulin signaling, namely, the PI3K/Akt pathway, which orchestrates most insulin metabolic actions, and the RAS/RAF/MAPK pathway, which regulates cell growth, survival and differentiation [28]. Several proteins in the RAS/RAF/MAPK pathway play a role in the development of the metabolic syndrome, through signaling kinases such as ERK1/2, JNK1 and p38s [29]. Mice with obesity show increased ERK1/2 activity, and its hepatic depletion improves systemic insulin action [30]. Hypothalamic JNK1 activation drives obesity and diabetes development in mice [31]. Finally, some p38s proteins promote hepatic IR and liver steatosis [32].

Dysregulation of PI3K/Akt signaling underlies several disorders including cancer, obesity, IR, metabolic-dysfunction-associated steatotic liver disease (MASLD) and T2D, either at the level of tissue inflammation or in the regulation of energy metabolism [33,34]. Surprisingly, both over- and under-activation of PI3K/Akt signaling appears to improve insulin sensitivity. For example, the absence of negative regulators of PI3K/Akt, such as PTP1B and PTEN (antagonists of insulin signaling), improves insulin sensitivity and protects against diet-induced IR in mice [35,36]; in contrast, systemic reduction in PI3K signaling by PTEN overexpression [37], or targeted inhibition of PI3K isoforms, increases energy expenditure and protects from obesity and metabolic syndrome [38,39]. The PI3K/Akt pathway is a key regulator of fetal growth [40], so it is likely that intrauterine epigenetic dysregulation of this pathway may occur as an adaptation to a nutrient-limited environment, facilitating fetal growth. In postnatal life, increased insulin sensitivity might be advantageous for short-term development, but detrimental for long-term health. This fetal programming of insulin pathways aligns well with the observed transition from increased insulin sensitivity at birth to subsequent IR in SGA infants [41,42,43]. The present results support previous data reporting that low BW children with a higher increase in the Z-score change from BW to BMI at age 7 yr display higher HOMA-IR and circulating IGF-1 and DHEAS levels, lower serum concentrations of HMW-adip and a higher liver fat fraction [44]. Circulating DHEAS concentrations, the marker of adrenarche, are directly associated with insulin, IGF-1, IR and adiposity [45], and an early and exaggerated adrenarche increases the risk of advanced puberty, PCOS and metabolic disturbances [45,46]. Therefore, it is possible that the upward mismatch between prenatal weight gain and postnatal weight gain leads to IR through a progressive impairment of PI3K/Akt and/or MAPK signaling, for example, in skeletal muscle [47,48]. A positive energy balance in postnatal life could also result in metabolic stress and adipose tissue dysfunction with increased lipolysis and a higher efflux of free fatty acids to the liver, which eventually evolves to MASLD [49,50]. Our results fit well with a previous study showing a strong association between IR and liver fat independently of BMI in prepubertal SGA children [10]; moreover, the SGA condition increases the risk of severe steatosis in children with obesity and MASLD [51].

Multivariate linear regression analysis uncovered that PCYOX1 and HSP90AA1, both up-regulated in cord-blood-derived exosomes of SGA infants, predicted liver fat at age 7 yr. PCYOX1 is involved in the degradation of prenylated proteins, mainly expressed by the liver and packaged into lipoproteins [52]. Increased PCYOX1 activity results in higher levels of the pro-atherogenic oxidized LDL, a novel biomarker of cardiovascular disease [52]. PCYOX1-deficient mice show less inflammation and lower lipid levels and peroxidation [53]. The key role of PCYOX1 in the regulation of adipogenesis both in vivo and in vitro has recently been posited [54]. While direct evidence linking PCYOX1 to liver fat is lacking, its role in oxidative stress, lipid metabolism and inflammation points to a role in hepatic steatosis. Along this line, mice lacking PCYOX1 and fed a high-fat diet show reduced body weight and visceral adipose depots [53]. HSP90AA1 is a highly conserved molecular chaperone involved in the folding, stabilization and maturation of essential proteins for normal cell functioning. Pharmacological inhibition of HSP90AA1 in mice with diabetes or diet-induced obesity and IR improves insulin sensitivity through the activation of HSF1, a key regulator of stress response [55]. Moreover, HSP90AA1 inhibition reduces liver fat in mice by regulating the stability and function of sterol regulatory element-binding protein (SREBP) [56].

The strengths of the present study include the strict inclusion criteria, avoiding overlap between study subgroups, and the longitudinal design, allowing the exploration of the associations of cord-blood-derived exosome proteins with developmental trajectories and metabolic outcomes from birth to age 7 yr. The limitations include the lack of SGA children without catch-up for comparisons, and the intrinsic drawbacks of label-free proteomics to detect low-abundance proteins with potential signaling functions. We are aware that the reported results require confirmation in larger and ethnically more heterogeneous cohorts of AGA and SGA subjects. This would facilitate the selection of the most relevant proteins for validation by Western blotting, which in turn, would strengthen these findings.

In conclusion, at birth, the proteome of circulating exosomes differs between AGA infants and SGA infants with spontaneous catch-up growth. A selection of DEPs at birth may become helpful to anticipate IR, adrenarche and/or ectopic adiposity in SGA children (with spontaneous catch-up) at age 7, when the early initiation of an insulin-sensitizing and/or ectopic-fat-reducing intervention could be considered.

## 4. Materials and Methods

### 4.1. Study Population

The initial study population consisted of 75 infants [40 AGA (48% girls) and 35 SGA (51% girls)], in whom the size and number of circulating exosomes at birth and at age 2 and 7 yr were previously assessed [15] (Flow-chart, Figure S6). These children were originally enrolled into one of two prospective longitudinal studies conducted at Hospital Sant Joan de Déu, Barcelona [15,16,17] (Flow-chart, Figure S6). As described, the inclusion criteria were the following: uncomplicated, singleton, term (37–42 weeks) pregnancy; AGA (BW between −1.0 SD and +1.0 SD) [15,16,17] or SGA at delivery (BW ≤ 2.0 SD) [14]; spontaneous catch-up in weight and length in SGA subjects (both >−2.0 SD by age 1 yr) [14]; exclusive breast or formula feeding for at least 4 months; and written informed consent. Exclusion criteria were maternal metabolic diseases, alcohol or drug abuse, complications at birth and evidence of congenital malformations.

All subjects had longitudinal data from birth to age 7 yr including auxology (height, weight, Tanner stage) and endocrine–metabolic parameters (glucose, insulin, IGF-1) at birth and at age 2 yr and 7 yr; body composition [by dual-energy X-ray absorptiometry (DXA)] at age 15 days and 2 yr; and abdominal fat distribution (subcutaneous, visceral and hepatic fat, by MRI) at age 7 yr.

Proteomic analysis of cord-blood-derived exosomes was performed in a subset of AGA (n = 20) and SGA (n= 20) subjects in whom complete longitudinal data and sufficient blood samples were available, which allowed for a uniform distribution by sex in both study subgroups (Flow-chart, Figure S6). No differences were detected in the proteome of cord-blood-derived exosomes between boys and girls in either AGA or SGA subgroup.

### 4.2. Clinical, Endocrine–Metabolic, Body Composition and Body Fat Distribution Assessments

Gestational age was calculated according to the last menses and confirmed by first-trimester ultrasound. Weight and length were measured after delivery; weight and height were measured again at age 2 yr and 7 yr, and BMI and Z-scores were derived [57].

Physical examination at age 7 yr confirmed that all children were still prepubertal (stage I by Tanner standards). Blood samples were obtained at birth from the umbilical cord before placental separation, and in the morning, in the fasting state at age 2 yr and 7 yr, as described [15].

Serum glucose was measured by the glucose-oxidase method. Insulin, IGF-1 and DHEA-S were assessed by imunochemiluminiscence (DPC IMMULITE 2500, Siemens, Erlangen, Germany). HOMA-IR was calculated as fasting insulin (mU/L) × fasting glucose (mmol/L)/22.5. HMW-adip was measured with a specific human enzyme-linked immunosorbent assay kit (R&D systems, Minneapolis, MN, USA). The intra- and inter-assay coefficients of variation (CVs) were <9%.

Body composition was assessed by DXA with a Lunar Prodigy coupled to Lunar software, version 3.4/3.5 (Lunar Corp., Madison, WI, USA). CVs were <3% for fat and lean mass [15,57,58]. Abdominal fat partitioning (subcutaneous and visceral fat areas) and the percentage of liver fat were assessed at the age of 7 yr by MRI, as described [59].

### 4.3. Exosome Isolation from Cord Blood

Cord blood exosomes were isolated and characterized as previously described [15]. Briefly, exosomes were isolated from cord blood using a modified protocol of the miRCURY exosome serum/plasma kit (Qiagen, Hilden, Germany) and characterized by Nanoparticle Tracking Analysis (NTA), Transmission Electron Microscopy (TEM), Scientific and Technological Centers (CCiTUB, University of Barcelona, Barcelona, Spain) and Western blotting [15], in accordance with the Minimal Information for Studies of Extracellular Vesicles (MISEV) criteria [60].

### 4.4. Proteomic Analysis of Cord-Blood-Derived Exosomes

#### 4.4.1. Protein Extraction and In-StageTip Digestion (iST)

Forty samples of isolated exosomes kept at −80 °C (n = 20 from AGA and n = 20 from SGA) were selected for label-free proteomic analysis, performed at the Proteomics Unit of the Universidad Complutense de Madrid. We incubated 50 µL of exosome-PBS samples in 50 µL of lysis buffer provided in the iST sample preparation kit (PreOmics GmbH, Planegg/Martinsried, Munich, Germany) for 10 min at 95 °C. Then, in-StageTip digestion was performed according to the manufacturer’s instructions (PreOmics). Briefly, lysated samples were loaded onto the stageTips, and digestion buffer was added. After 2 h of incubation at 37 °C, stop buffer was transferred to the samples. Afterwards, the devices were centrifuged at 3800× *g* for 2 min and washed twice using two different wash buffers, which were provided in the kit. The StageTips were then placed into fresh collection tubs, and peptides were recovered by centrifugation at 2800× *g* for 1 min using the elution buffer. The elution step was repeated once. Eluted peptides were vacuum-dried and reconstituted in LC-loading buffer.

#### 4.4.2. Liquid Chromatography with Tandem Mass Spectrometry (LC-MS/MS Analysis)

Liquid Chromatography with tandem mass spectrometry (LC-MS-MS) analyses were performed using a nano-UHPLC system (Vanquish Neo, Thermofisher scientific, Waltham, MA, USA), which was coupled online to a Q-exactive HF high-definition mass spectrometer (Thermofisher scientific). Tryptic digests corresponding to 1 µg of peptides were loaded directly into a trap column Acclaim PepMap 100, 2 cm × 75 µm i.d., 3 µm, 100 A (Thermofisher scientific) at 5 µL/min with aqueous solution containing 0.05% (*v*/*v*) trifluoroacetic acid and 1% acetonitrile. After 3 min, the trap column was set online with an analytical column Acclaim PepMap RSLC 50 cm × 75 µm i.d., C18, 2 µm, 100 A (Thermofisher scientific). Peptide elution was performed by applying a mixture of solvent A/B. Solvent A was HPLC-grade water with 0.1% (*v*/*v*) formic acid, and solvent B was HPLC-grade acetonitrile with 0.1% (*v*/*v*) formic acid. Peptides were separated running a gradient from 2 to 35% (*v*/*v*) mobile phase B at a flow rate of 250 nL/min over 90 min, followed by a gradient from 35 to 45% (*v*/*v*) mobile phase B over 10 min. Eluted samples were analyzed by Q-exactive HF (Thermofisher Scientific) using a Full MS/dd-MS^2^ mode. Data were obtained in the data-dependent acquisition mode at a scan range of 350–1800 *m*/*z*, and in the positive mode with a maximum injection time of 50 ms and an automatic gain control (AGC) value of 4.0 × 10^5^ using an Orbitrap mass analyzer at a mass resolution of 60.000. The top 15 intense precursor ions were selected for each duty cycle and subjected to higher energy collision-induced dissociation (HCD) with 20% normalized collision energy. The fragmented ions were detected using an Orbitrap mass analyzer at a resolution of 30.000 with a maximum injection time of 120 ms.

#### 4.4.3. Data Processing of LC-MS/MS Analysis

Mass spectrometry-derived data were searched against the UniprotKB/Swissprot human protein database (consisting of 20.307 entries) in Proteome Discoverer 2.2 (Thermofisher Scientific) using the Mascot (version 2.6.0; matrix science, London, UK) search algorithm. The parameters included trypsin as a proteolytic enzyme with a maximum of two missed cleavages. Cysteine carbamidomethylation was specified as fixed modification, and acetylation of the protein N-terminus and oxidation of methionine were set as variable modifications, with a minimum peptide length of seven amino acids. The precursor mass tolerance was fixed at 10 ppm, and 0.05 Da was set for fragment ion tolerance. The data were searched against a percolator algorithm with a 1% false discovery rate cutoff at the peptide level. Label-free relative quantification was performed using the precursor ion quantifier node and was based on the intensity of the unique peptide precursors from 90% of replicate features (Proteome Discoverer 2.2, ThermoFisher Scientific, Waltham, MA, USA). Normalization was performed on the total peptide amount. Protein abundances were calculated as the average of the most abundant distinct peptide groups, while the protein ratio was directly calculated from the grouped protein abundances. The statistical significance of the quantification ratio comparison was calculated by ANOVA. Label-free quantification values were log_2_ transformed, and the mean value within each group was used for further analysis. Proteins were considered differentially expressed between groups if the fold change (FC) was ≥1.5 or ≤0.5 and *p* < 0.05 (by an unpaired *t*-test).

### 4.5. Bioinformatics Analysis

#### 4.5.1. Gene Ontology (GO) Function and KEGG Pathway Analysis

To determine the involvement of the identified proteins in BPs and their molecular functions (MFs), along with the protein categories and CCs, an international standardized gene function classification system of GO (http://www.geneontology.org/, PANTHER version 18.0, accessed on 12 September 2024) was performed. The DAVID, https://david.ncifcrf.gov/, accessed on 16 September 2024) was used to perform KEGG pathway enrichment analysis.

#### 4.5.2. Protein–Protein Interaction (PPI) Network Analysis

The PPI network of target proteins was obtained from the Search Tool for the Retrieval of Interacting Genes (STRING V12.0; https://string-db.org/, accessed on 24 September 2024) [61] database, with the minimum required interaction score ≥ 0.7. The PPI network was visualized using Cytoscape v3.10.2. In the PPI network, nodes represent the target proteins, while edges represent the predicted or validated interaction between proteins. The molecular complex detection (MCODE) plug-in of Cytoscape was applied to detect subnetworks in the PPI network (k-core = 2, degree cutoff = 2, max. depth = 100 and node score cutoff = 0.2). Subsequently, KEGG pathway analysis of DEGs in modules was performed using the DAVID database.

### 4.6. Statistical Analysis and Ethics

Statistical analyses were performed using GraphPad Prism Software 8 (La Jolla, San Diego, CA, USA) and with SPSS Statistics 23.0 (IBM Corp., Armonk, NY, USA). Data are expressed as the mean ± SEM. Variables were checked for a normal distribution through the Kolmogorov–Smirnov test. For comparisons between groups, a two-tailed Student’s *t*-test was performed. Fisher’s test was used to compare categorical variables between groups. Correlation and stepwise multiple regression analysis were performed to assess the associations between cord-blood-derived exosomal proteins and auxological, metabolic and body composition parameters, and with abdominal fat partitioning and liver fat. Receiver operating characteristic (ROC) curves were plotted for each protein belonging to the PPI network constructed using the STRING database to assess their potential for discriminating low from normal birth weights. Only those proteins with an AUC > 0.8 were selected. The level of significance was set at *p* < 0.05.

## Figures and Tables

**Table 1:** Differentially expressed cord-blood-derived exosome proteins involved in KEGG and Reactome pathways plausibly related to the condition of being born small for gestational age (SGA). Pathways are arranged according to their *p*-value (from top to bottom).

Category	Term	Count	*p* Value
KEGG_PATHWAY	Complement and coagulation cascades	10	1.6 × 10^−9^
KEGG_PATHWAY	Phagosome	8	3.1 × 10^−5^
KEGG_PATHWAY	ECM–receptor interaction	6	1.8 × 10^−4^
KEGG_PATHWAY	Lipid and atherosclerosis	6	9.1 × 10^−3^
KEGG_PATHWAY	Regulation of actin cytoskeleton	7	2.4 × 10^−3^
KEGG_PATHWAY	PI3K-Akt signaling pathway	7	2.0 × 10^−2^
KEGG_PATHWAY	Tight junction	5	1.9 × 10^−3^
KEGG_PATHWAY	Focal adhesion	7	1.3 × 10^−3^
KEGG_PATHWAY	Efferocytosis	4	6.7 × 10^−2^
REACTOME_PATHWAY	Innate immune system	26	5.8 × 10^−9^
REACTOME_PATHWAY	Hemostasis	16	8.9 × 10^−6^
REACTOME_PATHWAY	Extracellular matrix organization	10	1.4 × 10^−4^
REACTOME_PATHWAY	Nervous system development	12	1.2 × 10^−3^
REACTOME_PATHWAY	Metabolism of proteins	25	2.3 × 10^−3^
REACTOME_PATHWAY	Vesicle-mediated transport	12	4.0 × 10^−3^
REACTOME_PATHWAY	Signal transduction	27	1.1 × 10^−2^
REACTOME_PATHWAY	Membrane trafficking	9	5.3 × 10^−2^
REACTOME_PATHWAY	Cell–cell communication	4	7.8 × 10^−2^

**Table 2:** Multivariate linear model of liver fat at age 7 years and selected proteomic and endocrine–metabolic parameters.

	Liver Fat at Age 7 Years		
	Beta	Significance	R^2^
PCYOX1	0.432	0.007	0.328
HSP90AA1	0.689	0.005	0.377
IGF-1 at age 2 years	0.465	0.017	0.110

Non-predictive variables: total fat at age 2 yr, ORM2, APOC4, SERPING1, SNCA, MMP9, GP5. PCYOX1, prenylcysteine oxidase 1; HSP90AA1, Heat Shock Protein 90 Alpha Family Class A Member 1; IGF-1, insulin-like growth factor-1.

## Data Availability

Data have been deposited in the Zenodo repository as supporting data values, DOI: 10.5281/zenodo.13847363, and are publicly available as of the date of publication.

## Supplementary Materials

The following supporting information can be downloaded at https://www.mdpi.com/article/10.3390/ijms26041721/s1.
