# Supplementary material for: The Proteome of Exosomes at Birth Predicts Insulin Resistance, Adrenarche and Liver Fat in Childhood
Source: Int J Mol Sci. 2025 Feb 18;26(4):1721. doi: 10.3390/ijms26041721 (PMC11854951; doi:10.3390/ijms26041721)
Supplement: Supplementary file 1 [file ijms-26-01721-s001.zip › ijms-3458930-supplementary.pdf]

Table S1. Longitudinal data (0-7 yr) in infants born appropiate-for-gestational-age (AGA, n=20) or small-for-gestational-age (SGA, n=20)

	At birth		2 yr		7 yr	
	AGA	SGA	AGA	SGA	AGA	SGA
**Auxology**						
Gestational age (wk)	**39.5 ± 0.3**	**38.4 ± 0.4***	--	--	--	--
Weight SDS	**-0.1 ± 0.1**	**-2.3 ± 0.1*****	0.1 ± 0.3	-0.4 ± 0.4	0.3 ± 0.4	0.1 ± 0.3
Length SDS	**-0.3 ± 0.2**	**-1.7 ± 0.1*****	-0.4 ± 0.4	0.1 ± 0.4	0.5 ± 0.3	-0.1 ± 0.3
BMI (Kg/m^2)	**13.5 ± 0.2**	**10.7 ± 0.1*****	16.6 ± 0.4	15.4 ± 0.4	16.8 ± 0.6	17.1 ± 0.7
Δ BMI-BW SDS	--	--	**0.3 ± 0.4**	**1.6 ± 0.4***	**0.1 ± 0.3**	**2.4 ± 0.3*****
Sex (% females)	55	45				
Cesarean section (%)	10	35				
**Endocrine-metabolic variables**						
HOMA-IR	--	--	0.8 ± 0.3	0.7 ± 0.2	**0.6 ± 0.1**	**1.2 ± 0.2****
IGF-1 (nmol/L)	**7.7 ± 0.7**	**4.8 ± 0.4****	9.9 ± 1.3	11.0 ± 1.3	**19.2 ± 1.5**	**28.6 ± 2.8****
HMW-adip (mg/L)	**28.2 ± 2.1**	**20.6 ± 2.0***	15.5 ± 3.1	17.4 ± 2.6	**14.9 ± 1.7**	**8.8 ± 1.2****
DHEAS (µmol/L)	--	--	--	--	**0.9 ± 0.2**	**2.1 ± 0.6***
SHBG (nmol/L)	--	--	--	--	**109 ± 9**	**90 ± 8**
**Body Composition (DXA)**						
BMD (g/cm^2)	**0.24 ± 0.01**	**0.20 ± 0.01*****	0.47 ± 0.01	0.48 ± 0.02	--	--
BMC (g)	**101.4 ± 2.9**	**84.2 ± 3.5*****	440 ± 18	474 ± 27	--	--
Fat mass (Kg)	**0.73 ± 0.05**	**0.46 ± 0.04*****	4.1 ± 0.2	3.7 ± 0.3	--	--
Abdominal fat (Kg)	**0.039 ± .004**	**0.026 ± .002****	0.19 ± 0.01	0.17 ± 0.01	--	--
Lean mass (Kg)	**3.0 ± 0.1**	**2.4 ± 0.1*****	8.5 ± 0.3	8.1 ± 0.4	--	--
**MRI**						
Subcutaneous fat (cm^2)	--	--	--	--	41.8 ± 8.1	52.1 ± 10.6
Visceral fat (cm^2)	--	--	--	--	16.6 ± 1.8	17.3 ± 1.9
Liver volume (mL)	--	--	--	--	732 ± 28	706 ± 26
Liver fat (%)	--	--	--	--	**11.5 ± 1.1**	**15.4 ± 1.3***

BMI, body mass index; BW, birth weight; HOMA-IR, homeostasis model assessment-insulin resistance; IGF-1, insulin-like growth factor-1;

HMW-adip,high-molecular-weight adiponectin; DHEAS, dehydroepiandrosterone sulfate; DXA, dual energy X-ray absorptiometry; BMD, bone mineral density;

BMC, bone mineral content; MRI, magnetic resonance imaging.

Values are mean ± SEM. *P<0.05, **P<0.01, *** P<0.001 *vs* AGA.

Significant values are highlighted in bold.

Figure S1. Representative nanoparticle-tracking analysis (NTA) of isolated exosomes from cord blood in infants born appropiate- (AGA) or small-for-gestational-age (SGA)

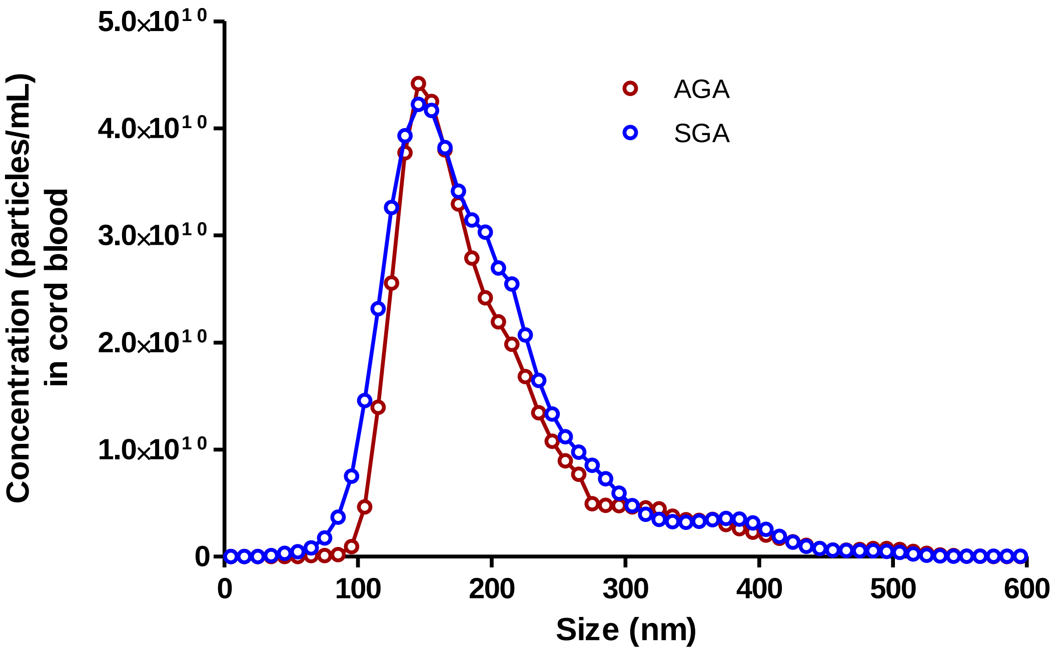

Table S2A. Proteins exclusively identified in cord blood-derived exosomes from appropriate-for-gestational-age (AGA, n=20)

Protein accession	Protein name	Abbreviations
Q13813	Spectrin alpha chain, non-erythrocytic 1	SPTAN1
P53396	ATP-citrate synthase	ACLY
Q03591	Complement factor H-related protein 1	CFHR1
P04083	Annexin A1	ANXA1
Q15166	Serum paraoxonase/lactonase 3	PON3
P78371	T-complex protein 1 subunit beta	CCT2
A0A0G2JMI3	Immunoglobulin heavy variable 1-69-2	IGHV1-69-2
P07437	Tubulin beta chain	TUBB
P62258	14-3-3 protein epsilon	YWHAE
A0A0C4DH32	Immunoglobulin heavy variable 3-20	IGHV3-20
P00918	Carbonic anhydrase 2	CA2
P09960	Leukotriene A-4 hydrolase	LTA4H
Q86VP6	Cullin-associated NEDD8-dissociated protein 1	CAND1
P01763	Immunoglobulin heavy variable 3-48	IGHV3-48
P37837	Transaldolase	TALDO1
A0A075B6K5	Immunoglobulin lambda variable 3-9	IGLV3-9
P61224	Ras-related protein Rap-1b	RAP1B
A0A0B4J1X8	Immunoglobulin heavy variable 3-43	IGHV3-43
P11465	Pregnancy-specific beta-1-glycoprotein 2	PSG2
P07359	Platelet glycoprotein Ib alpha chain	GP1BA
Q9NPG4	Protocadherin-12	PCDH12
A0A0C4DH41	Immunoglobulin heavy variable 4-61	IGHV4-61
P07738	Bisphosphoglycerate mutase	BPGM
P62820	Ras-related protein Rab-1A	RAB1A
P01824	Immunoglobulin heavy variable 4-39	IGHV4-39
P15311	Ezrin	EZR
P40227	T-complex protein 1 subunit zeta	CCT6A
P43490	Nicotinamide phosphoribosyltransferase	MPT
P15153	Ras-related C3 botulinum toxin substrate 2	RAC2
A0A0C4DH67	Immunoglobulin kappa variable 1-8	IGKV1-8
P30044	Peroxiredoxin-5, mitochondrial	PRDX5
Q9H4M9	EH domain-containing protein 1	EHD1
P22891	Vitamin K-dependent protein Z	PROZ
P40121	Macrophage-capping protein	CAPG
P12956	X-ray repair cross-complementing protein 6	XRCC6
Q8IUL8	Cartilage intermediate layer protein 2	CILP2
Q15149	Plectin	PLEC
P20908	Collagen alpha-1(V) chain	COL5A1
P30566	Adenylosuccinate lyase OS=Homo sapiens OX=9606 GN=ADSL PE=1 SV=2	ADSL
Q9H4B7	Tubulin beta-1 chain	TUBB1

P01137	Transforming growth factor beta-1 proprotein	TGFB1
Q6YHK3	CD109 antigen	CD109
O00299	Chloride intracellular channel protein 1	CLIC1
P18065	Insulin-like growth factor-binding protein 2	IGFBP2
Q92859	Neogenin	NEO1
Q08830	Fibrinogen-like protein 1	FGL1
P16452	Protein 4.2	EPB42
Q9Y5Y7	Lymphatic vessel endothelial hyaluronic acid receptor 1	LYVE1
Q16851	UTP--glucose-1-phosphate uridylyltransferase	UGP2
P01236	Prolactin	PRL
Q9Y3I1	F-box only protein 7	FBXO7
Q16181	Septin-7	SEPTIN7
Q9NPH3	Interleukin-1 receptor accessory protein	IL1RAP
O43242	26S proteasome non-ATPase regulatory subunit 3	PSMD3
Q86UD1	Out at first protein homolog	OAF
P67775	Serine/threonine-protein phosphatase 2A catalytic subunit alpha isoform	PPP2CA
P06748	Nucleophosmin	NPM1
Q15080	Neutrophil cytosol factor 4	NCF4
Q8NDA2	Hemicentin-2	HMCN2

Table S2B. Proteins exclusively identified in cord blood-derived exosomes from small-for-gestational-age (SGA, n=20)

Protein accession	Protein name	Abbreviations
P11277	Spectrin beta chain, erythrocytic	SPTB
Q9UK55	Protein Z-dependent protease inhibitor	SERPI10
P46940	Ras GTPase-activating-like protein IQGAP1	IQGAP1
P13647	Keratin, type II cytoskeletal 5	KRT5
P13798	Acylamino-acid-releasing enzyme	APEH
P09172	Dopamine beta-hydroxylase	DBH
P0DML2	Chorionic somatomammotropin hormone 1	CSH1
P0DP08	Immunoglobulin heavy variable 4-38-2	IGHV4-38-2
P27105	Stomatin	STOM
P08567	Pleckstrin	PLEK
P61204	ADP-ribosylation factor 3	ARF3
P28066	Proteasome subunit alpha type-5	PSMA5
P15531	Nucleoside diphosphate kinase A	NME1
A0A0C4DH72	Immunoglobulin kappa variable 1-6	IGKV1-6
O15511	Actin-related protein 2/3 complex subunit 5	ARPC5
O75015	Low affinity immunoglobulin gamma Fc region receptor III-B	FCGR3B
P49368	T-complex protein 1 subunit gamma	CCT3
P06732	Creatine kinase M-type	CKM
P08397	Porphobilinogen deaminase	HMBS
Q96QK1	Vacuolar protein sorting-associated protein 35	VPS35
P48643	T-complex protein 1 subunit epsilon	CCT5
O00533	Neural cell adhesion molecule L1-like protein	CHL1
P24158	Myeloblastin	PRTN3
P04899	Guanine nucleotide-binding protein G(i) subunit alpha-2	GI2
Q99436	Proteasome subunit beta type-7	PSMB7
P05121	Plasminogen activator inhibitor 1	SERPINE1
P62333	26S proteasome regulatory subunit 10B	PSMC6
P20774	Mimecan	OGN
P22894	Neutrophil collagenase	MMP8
Q9UNS2	COP9 signalosome complex subunit 3	COPS3
P37802	Transgelin-2	TAGLN2
Q92905	COP9 signalosome complex subunit 5	COPS5
P07237	Protein disulfide-isomerase	P4HB
P11047	Laminin subunit gamma-1	LAMC1
P08123	Collagen alpha-2(I) chain	COL1A2
P0DSN7	Probable non-functional immunoglobulinn kappa variable 1D-37	IGKV1D-37
Q9UIA9	Exportin-7	XPO7
Q8NI99	Angiopoietin-related protein 6	ANGPTL6
P01033	Metalloproteinase inhibitor 1	TIMP1
P04839	Cytochrome b-245 heavy chain	CYBB

Table S3. Differentially expressed proteins in cord blood-derived exosomes from appropriate-for-gestational-age (AGA, n=20) and small-for-gestational-age (SGA, n=20) infants

Protein accession	Protein name	Abbreviations	Function	log_2 FC	*P* value	Adjusted† *P* value
P13591	Neural cell adhesion molecule 1	NCAM1	Involved in fetal growth. Regulation of formation and maintenance of synapses. Biomarker of coronary artery disease	1.5850	0.0001	0.0110
Q9UGM5	Fetuin-B	FETUB	Related to obesity, metabolic syndrome and non-alcoholic fatty liver disease	1.2332	0.0001	0.0055
Q9NZP8	Complement C1r subcomponent-like protein	C1RL	Associated to blood glucose and lipids and body fat	1.0580	0.0001	0.0037
P19652	Alpha-1-acid glycoprotein 2	ORM2	Acute phase-globulin. Associated with adiponectin, adipose tissue insulin resistance and type 2 diabetes	0.9318	0.0001	0.0028
P27797	Calreticulin	CALR	Interacts with the DNA-binding domain of NR3C1 and mediates its nuclear export; involved in maternal gene expression regulation	0.6210	0.0001	0.0022
Q01082	Spectrin beta chain, non-erythrocytic 1	SPTBN1	Central nervous system development and function. Candidate biomarker for childhood obesity	-1.0361	0.0001	0.0018
P12814	Alpha-actinin-1	ACTN1	Key regulator of cell motility, morphology and adhesion. May be involved in the development of obesity	-1.4056	0.0001	0.0016
P37840	Alpha-synuclein	SNCA	Prevents insulin resistance increasing glucose uptake in muscle and adipocytes	-1.4713	0.0001	0.0014
A0A075B6R2	Immunoglobulin heavy variable 4-4	IGHV4-4	Antigen recognition	-1.5407	0.0001	0.0012
O15144	Actin-related protein 2/3 complex subunit 2	ARPC2	Regulation of gene transcription and repair of DNA damage	-1.8033	0.0001	0.0011
Q9UBW5	Bridging integrator 2	BIN2	Podosome formation; inhibition of phagocytosis	-1.9945	0.0001	0.0010
P33908	Mannosyl-oligosaccharide 1,2-alpha-mannosidase IA	MAN1A1	Protein glycosylation	-2.0056	0.0001	0.0009
P14780	Matrix metalloproteinase-9	MMP9	Associated with type 2 diabetes and pathogenesis of diabetes complications	-2.2168	0.0001	0.0008
Q8IUI8	Cytokine receptor-like factor 3	CRLF3	Critical regulator of neurogenesis, neuroprotective EV-3 (Epo) receptor	-4.5956	0.0001	0.0008
Q14515	SPARC-like protein 1	SPARCL1	Regulation of IGF1 transport and uptake by IGFBPs. Involved in non-alcoholic steatohepatitis progression	4.5401	0.0002	0.0015
Q99715	Collagen alpha-1(XII) chain	COL12A1	Plays a role in obesity	2.6133	0.0002	0.0014
P68104	Elongation factor 1-alpha 1	EEF1A1	Critical player in lipotoxic cell death. Involved in diabetic cardiomyopathy	1.3370	0.0003	0.0019
P05155	Plasma protease C1 inhibitor	SERPING1	Inhibition of the complement system. Associated with the development of type 2 diabetes	-1.8832	0.0004	0.0024
P00740	Coagulation factor IX	F9	Role in type 2 diabetes and cardiovascular disease	1.1550	0.0005	0.0029
P35579	Myosin-9	MYH9	Role in cytokinesis, cell migration, polarization and adhesion. Tumor suppressor gene	-1.1495	0.0006	0.0033
P04430	Immunoglobulin kappa variable 1-16	IGKV1-16	Antigen recognition	1.2275	0.0007	0.0037
P18428	Lipopolysaccharide-binding protein	LBP	Role in lipid metabolism and cardiovascular diseases	-1.1756	0.0007	0.0035
P20700	Lamin-B1	LMNB1	Crucial role in nuclear function and organization. Plays a role in the etiology of MASLD	2.8520	0.0010	0.0048
Q9BXJ4	Complement C1q tumor necrosis factor-related protein 3	C1QTNF3	Associated to type 2 diabetes and coronary artery disease	1.9332	0.0010	0.0046
Q9Y6Z7	Collectin-10	COLEC10	Recognition molecule in the lectin complement pathway. Mediator of host defense and maintenance of tissue homeostasis	1.4284	0.0010	0.0044
P55056	Apolipoprotein C-IV	APOC4	Has a role in lipid metabolism. Variants in this gene are associated with hypertriglyceridemia, coronary artery disease and type 2 diabetes	1.1980	0.0010	0.0042
A0A075B6H7	Probable non-functional immunoglobulin kappa variable 3-7	IGKV3-7	Associated with osteoporosis	0.5859	0.0010	0.0041
P07900	Heat shock protein HSP 90-alpha	HSP90AA1	Related to lipid metabolism. Involved in MASLD progression	0.8659	0.0011	0.0043
P08571	Monocyte differentiation antigen CD14	CD14	Associated with obesity and insulin resistance	1.2758	0.0014	0.0053
P02753	Retinol-binding protein 4	RBP4	Associated with obesity, type 2 diabetes and cardiovascular disease	0.6465	0.0014	0.0051

Table S3 (Continued). Differentially expressed proteins in cord blood-derived exosomes from appropriate-for-gestational-age (AGA, n=20) and small-for-gestational-age (SGA, n=20) infants

Protein accession	Protein name	Abbreviations	Function	log_2 FC	*P* value	Adjusted* *P* value
P00450	Ceruloplasmin	CP	New adipokine associated with obesity and obesity-associated cancers	-1.0799	0.0018	0.0064
P02042	Hemoglobin subunit delta	HBD	Related to beta-thalassemia. Confers protection against oxidative stress	-1.4500	0.0019	0.0065
P01619	Immunoglobulin kappa variable 3-20	IGKV3D-20	Antigen recognition	0.7423	0.0020	0.0067
P02549	Spectrin alpha chain, erythrocytic 1	SPTA1	Associated with hypertension. Involved in red cell membrane disorders	-2.1753	0.0020	0.0065
Q07954	Prolow-density lipoprotein receptor-related protein 1	LRP1	Regulation of insulin signaling and glucose uptake. Regulation of food intake and energy expenditure	1.0562	0.0029	0.0091
P11717	Cation-independent mannose-6-phosphate receptor	IGF2R	Growth and development, clearance of IGF2, lysosomal enzyme transport. Has a role in neurotransmission and in memory enhancement/consolidation	1.1125	0.0030	0.0092
P07360	Complement component C8 gamma chain	C8G	Associated with blood glucose and lipids and with body fat	0.8267	0.0030	0.0089
P04908	H2A Clustered Histone 4	H2AC4	Nucleosome structure	-2.7743	0.0030	0.0087
P11226	Mannose-binding protein C	MBL2	Involved in immune system function, inflammation and insulin action	0.8922	0.0035	0.0099
P01743	Immunoglobulin heavy variable 1-46	IGHV1-46	Antigen recognition	1.8647	0.0040	0.0110
P04275	von Willebrand factor	VWF	Has a role in type 2 diabetes and cardiovascular diseases	0.8040	0.0040	0.0107
P25311	Zinc-alpha-2-glycoprotein	AZGP1	Lipolytic adipokine implicated in regulation of adipose tissue metabolism and fat distribution. Associated with insulin resistance and type 2 diabetes	0.8739	0.0049	0.0128
P35908	Keratin, type II cytoskeletal 2 epidermal	KRT2	Critical role in the cytoskeletal network	0.9249	0.0050	0.0128
Q15848	Adiponectin	ADIPOQ	Has a role in obesity, type 2 diabetes , endothelial dysfunction and inflammation	0.8386	0.0050	0.0125
P22692	Insulin-like growth factor-binding protein 4	IGFBP4	IGFs binding protein	0.6802	0.0050	0.0122
P05090	Apolipoprotein D	APOD	Key factor in the development of obesity and type 2 diabetes	-0.5528	0.0050	0.0120
Q9UHG3	Prenylcysteine oxidase 1	PCYOX1	Key regulator of adipogenesis. Plays a role in atherosclerosis	1.3933	0.0056	0.0131
P01024	Complement C3	C3	Contributes to the incidence of type 2 diabetes. Associated with β-cell function	0.5889	0.0057	0.0131
A0A075B6I1	Immunoglobulin lambda variable 4-60	IGLV4-60	Antigen recognition	1.2597	0.0060	0.0135
P13645	Keratin, type I cytoskeletal 10	KRT10	Critical role in the cytoskeletal network	1.1427	0.0060	0.0132
O14786	Neuropilin-1	NRP1	Has a role in obesity and metabolic syndrome	0.9534	0.0060	0.0129
A0A075B6K4	Immunoglobulin lambda variable 3-10	IGLV3-10	Antigen recognition	0.8789	0.0060	0.0127
A0A075B6J9	Immunoglobulin lambda variable 2-18	IGLV2-18	Antigen recognition	0.7906	0.0060	0.0125
P07996	Thrombospondin-1	THBS1	Adipokine associated with obesity, adipose tissue inflammation and insulin resistance	0.5946	0.0060	0.0122
A0A0C4DH25	Immunoglobulin kappa variable 3D-20	IGKV3D-20	Antigen recognition	0.5933	0.0060	0.0120
Q4LDE5	Sushi, von Willebrand factor type A, EGF and pentraxin domain-containing protein 1	SVEP1	Extracellular matrix protein related to cardiovascular disease, hypertension and type 2 diabetes	-1.4468	0.0060	0.0118
P31146	Coronin-1A	CORO1A	Involve in cytokinesis, cell motility, immune homeostasis and calcium-calcineurin signaling	1.1654	0.0070	0.0135
P0C0L5	Complement C4-B	C4B	Associated with type 2 diabetes	0.7362	0.0072	0.0137
P40197	Platelet glycoprotein V	GP5	Associated with type 2 diabetes	-1.3078	0.0079	0.0147
P29622	Kallistatin	SERPINA4	Protective role against oxidative stress and inflammation	0.4637	0.0090	0.0165

Table S3 (Continued). Differentially expressed proteins in cord blood-derived exosomes from appropriate-for-gestational-age (AGA, n=20) and small-for-gestational-age (SGA, n=20) infants

Protein accession	Protein name	Abbreviations	Function	log_2 FC	*P* value	Adjusted* *P* value
Q15582	Transforming growth factor-beta-induced protein ig-h3	TGFBI	Associated with diabetes-risk and MASLD	0.3993	0.0090	0.0162
P33151	Cadherin-5	CDH5	Associated with MASLD in children	-0.6190	0.0090	0.0160
Q6EMK4	Vasorin	VASN	Maintenance of vascular function. Its deficiency leads to cardiac hypertrophy	-1.1675	0.0090	0.0157
P05556	Integrin beta-1	ITGB1	Key regulator of adipose tissue function and whole-body metabolism	-1.6737	0.0090	0.0155
P01130	Low-density lipoprotein receptor	LDLR	Orchestrates cholesterol homeostasis	1.6363	0.0094	0.0159
P06276	Cholinesterase	BCHE	Associated with obesity and metabolic syndrome prediction	-1.7277	0.0098	0.0163
P61586	Transforming protein RhoA	RHOA	Regulation of food intake and body weight	0.6440	0.0110	0.0181
P36871	Phosphoglucomutase-1	PGM1	Key regulator of glycogen metabolism	1.3721	0.0120	0.0194
P50991	T-complex protein 1 subunit delta	CCT4	Component of the chaperonin-containing T-complex (TRiC), a molecular chaperone complex that assists the folding of proteins	1.3692	0.0120	0.0191
P01594	Immunoglobulin kappa variable 1-33	IGKV1-33	Antigen recognition	0.6569	0.0120	0.0189
P05067	Amyloid-beta precursor protein	APP	Associated with obesity, type 2 diabetes, MASLD, cardiovascular diseases and cancer	1.2016	0.0130	0.0201
P50395	Rab GDP dissociation inhibitor beta	GDI2	Regulation of vesicle-mediated cellular transport. Has a role in the development of type 2 diabetes	-3.8421	0.0150	0.0229
P00491	Purine nucleoside phosphorylase	PNP	Key enzyme of the purine metabolism	1.3337	0.0160	0.0241
P01871	Immunoglobulin heavy constant mu	IGHM	Antigen recognition	1.2500	0.0170	0.0253
P24821	Tenascin	TNC	Associated with cardiovascular diseases and type 2 diabetes	0.9955	0.0190	0.0279
P02776	Platelet factor 4	PF4	Has a role in type 2 diabetes and in atherosclerosis	0.7353	0.0190	0.0275
P68032	Actin, alpha cardiac muscle 1	ACTC1	Component of cytoskeleton	-1.7058	0.0209	0.0299
P02538	Keratin, type II cytoskeletal 6A	KRT6A	Involved in wound healing	0.7289	0.0212	0.0299
P01031	Complement C5	C5	Associated with an increased risk and severity of MASLD and type 2 diabetes	0.8055	0.0234	0.0326
Q00887	Pregnancy-specific beta-1-glycoprotein 9	PSG9	Pro-angiogenic factor	1.1696	0.0250	0.0344
Q12860	Contactin-1	CNTN1	Regulation of myelin formation and organization of nodal and paranodal domains in the CNS	0.6669	0.0250	0.0340
P15144	Aminopeptidase N	ANPEP	Associated with MASLD and with the development of type 2 diabetes	0.8542	0.0260	0.0349
P20618	Proteasome subunit beta type-1	PSMB1	Involved in the ATP-dependent degradation of ubiquitinated proteins	0.8710	0.0270	0.0358
P49747	Cartilage oligomeric matrix protein	COMP	Regulation of adipogenesis. Its circulating levels correlate with obesity	0.5974	0.0280	0.0367
P02745	Complement C1q subcomponent subunit A	C1QA	Associated with hyperglycemia, hyperlipidemia and with β-cell function	0.9599	0.0310	0.0401
P63241	Eukaryotic translation initiation factor 5A-1	EIF5A	Contributes to the development of diabetes	1.2340	0.0320	0.0409
P05156	Complement factor I	CFI	Inhibition of all complement pathways	0.7048	0.0320	0.0405
Q13822	Ectonucleotide pyrophosphatase/phosphodiesterase family member 2	ENPP2	Associated with obesity and insulin resistance	1.8175	0.0350	0.0438
Q13201	Multimerin-1	MMRN1	Involved in atherosclerosis and myocardial infarction	1.2491	0.0350	0.0433
P0DOX6	Immunoglobulin mu heavy chain	IGM	Antigen recognition	1.1127	0.0397	0.0485
P63218	Guanine nucleotide-binding protein G(I)/G(S)/G(O) subunit gamma-5	GNG5	G protein acting as a modulator of transmembrane signal transduction	0.6490	0.0420	0.0508

NR3C1, nuclear receptor subfamily 3 group C member 1; IGFBPs, Insulin-like growth factor binding proteins; MASLD, metabolic dysfunction-associated steatotic liver disease; IGF2, insulin-like growth factor-2; IGFs, insulin-like growth factors.

†Obtained after adjustment for multiple testing by using the Benjamini-Hochberg procedure and a false discovery rate (FDR) cutoff of 0.05.

Table S4. Proteins not differentially expressed in cord blood-derived exosomes from appropriate-for-gestational-age (AGA, n=20) and small-for-gestational-age (SGA, n=20) infants

Protein name	Abbreviations	log_2 FC	*P* value
Glucose-6-phosphate isomerase	GPI	-1.3823	0.0640
Hemoglobin subunit mu	HBM	-1.1301	0.0700
Protein S100-A9	S100A9	-1.0976	0.2160
Actin-related protein 2/3 complex subunit 3	ARPC3	-0.9609	0.0780
Fibrinogen alpha chain	FGA	-0.9084	0.6920
Coagulation factor XIII A chain	F13A1	-0.8860	0.1260
Immunoglobulin heavy constant alpha	IGHA1	-0.8717	0.1990
L-selectin	SELL	-0.8013	0.1100
Immunoglobulin heavy variable 3-64D	IGHV3-64D	-0.7832	0.0860
Moesin	MSN	-0.7743	0.2540
Serpin A11	SERPINA11	-0.7482	0.0720
Profilin-1	PFN1	-0.7054	0.1410
Flavin reductase (NADPH)	BLVRB	-0.6915	0.2140
Hemoglobin subunit zeta	HBZ	-0.6734	0.2830
Fibrinogen beta chain	FGB	-0.6349	0.4370
Pappalysin-1	PAPPA	-0.6298	0.0690
Coagulation factor XII	F12	-0.6155	0.0790
Keratin. type II cuticular Hb1	KRT81	-0.5984	0.1420
Immunoglobulin lambda variable 7-43	IGLV7-43	-0.5937	0.2250
Alpha-1B-glycoprotein	A1BG	-0.5359	0.1120
Apolipoprotein L1	APOL1	-0.5317	0.1610
Myosin light polypeptide 6	MYL6	-0.5209	0.3750
Immunoglobulin heavy variable 1-2	IGHV1-2	-0.5162	0.0810
Fibrinogen gamma chain	FGG	-0.5111	0.7560
Protein kinase C-binding protein NELL2	NELL2	-0.5082	0.0720
Thioredoxin	TXN	-0.5046	0.2560
Ubiquitin-40S ribosomal protein S27a	RPS27A	-0.5042	0.0760
Hyaluronan-binding protein 2	HABP2	-0.4951	0.0740

Protein-glutamine gamma-glutamyltransferase 2	TGM2	-0.4748	0.1180
Peptidyl-prolyl cis-trans isomerase A	PPIA	-0.4659	0.2360
Multiple epidermal growth factor-like domains protein 8	MEGF8	-0.4649	0.3850
Protein S100-P	S100P	-0.4643	0.4120
Heat shock cognate 71 kDa protein	HSPA8	-0.4580	0.6970
Immunoglobulin lambda variable 3-27	IGLV3-27	-0.4577	0.0590
Coagulation factor X	F10	-0.4558	0.0890
Cathepsin	CTSG	-0.4558	0.4480
Ficolin-1	FCN1	-0.4477	0.0950
Sex hormone-binding globulin	SHBG	-0.4428	0.2660
Noelin	OLFM1	-0.4372	0.0960
Immunoglobulin heavy variable 4-30-2	IGHV4-30-2	-0.4302	0.0680
Dermokine	DMKN	-0.4167	0.1160
Serum paraoxonase/arylesterase 1	PON1	-0.4087	0.2120
Periostin	POSTN	-0.4058	0.2240
Alpha-1-antichymotrypsin	SERPINA3	-0.4043	0.1340
Histidine-rich glycoprotein	HRG	-0.4034	0.0850
Brain acid soluble protein 1	BASP1	-0.3930	0.3470
Tropomyosin alpha-4 chain	TPM4	-0.3918	0.3410
Alpha-1-antitrypsin	SERPINA1	-0.3332	0.0580
L-lactate dehydrogenase B chain	LDHB	-0.3813	0.0560
Immunoglobulin heavy variable 3-72	IGHV3-72	-0.3750	0.0760
Fructose-bisphosphate aldolase A	ALDOA	-0.3663	0.2330
Apolipoprotein A-II	APOA2	-0.3603	0.0610
Galectin-3-binding protein	LGALS3BP	-0.3542	0.1670
Hemoglobin subunit alpha	HBA1	-0.3537	0.2480
Histone H3.1	H3C1	-0.3450	0.6620
Hepatocyte growth factor-like protein	MST1	-0.3344	0.0820
Catalase	CAT	-0.3296	0.5670
Corticosteroid-binding globulin	SERPINA6	-0.3288	0.2060
Glutathione peroxidase 3	GPX3	-0.3286	0.1430
Protein disulfide-isomerase A3	PDIA3	-0.3245	0.3510

EGF-containing fibulin-like extracellular matrix protein 1	EFEMP1	-0.3206	0.5530
Immunoglobulin heavy variable 3-9	IGHV3-9	-0.3188	0.3420
Transitional endoplasmic reticulum ATPase	VCP	-0.3188	0.7240
Actin. cytoplasmic 1	ACTB	-0.3172	0.5200
14-3-3 protein gamma	YWHAG	-0.3170	0.3890
Cofilin-1	CFL1	-0.3033	0.3940
Collagen alpha-3(VI) chain	COL6A3	-0.2913	0.1890
Delta-aminolevulinic acid dehydratase	ALAD	-0.2822	0.7350
Immunoglobulin heavy variable 3-73	IGHV3-73	-0.2817	0.5190
Immunoglobulin lambda variable 3-19	IGLV3-19	-0.2749	0.4200
Immunoglobulin gamma-1 heavy chain	IGG1	-0.2704	0.1270
Apolipoprotein A-IV	APOA4	-0.2696	0.1430
Filamin-A	FLNA	-0.2682	0.3040
Adenylyl cyclase-associated protein 1	CAP1	-0.2651	0.7160
WD repeat-containing protein 1	WDR1	-0.2544	0.0750
Alpha-2-macroglobulin	A2M	-0.2464	0.0976
Plasma serine protease inhibitor	SERPINA5	-0.2426	0.4810
Apolipoprotein A-I	APOA1	-0.2355	0.1130
Glycogen phosphorylase. liver form	PYGL	-0.2310	0.6360
Phosphatidylcholine-sterol acyltransferase	LCAT	-0.2223	0.5370
Collagen alpha-2(V) chain	COL5A2	-0.2202	0.4920
Cystatin-C	CST3	-0.2117	0.5560
Polymeric immunoglobulin receptor	PIGR	-0.2080	0.3340
Carboxypeptidase N catalytic chain	CPN1	-0.2040	0.1510
Ficolin-2	FCN2	-0.1964	0.7790
Complement C2	C2	-0.1964	0.2200
Serotransferrin	TF	-0.1847	0.5650
Prostaglandin-H2 D-isomerase	PTGDS	-0.1837	0.4060
Plasma kallikrein	KLKB1	-0.1837	0.2870
Kininogen-1	KNG1	-0.1771	0.3300
Clusterin	CLU	-0.1767	0.2770
Inter-alpha-trypsin inhibitor heavy chain H3	ITIH3	-0.1688	0.2540
Attractin	ATRN	-0.1644	0.5300

Albumin	ALB	-0.1633	0.1560
Apolipoprotein C-III	APOC3	-0.1604	0.4940
Selenoprotein P	SELENOP	-0.1423	0.5720
Peroxiredoxin-2	PRDX2	-0.1403	0.8250
Alpha-2-antiplasmin	SERPINF2	-0.1361	0.2950
HLA class I histocompatibility antigen. A alpha chain	HLA-A	-0.1317	0.8840
Platelet basic protein	PPBP	-0.1302	0.4580
Triosephosphate isomerase	TPI1	-0.1225	0.8030
Adipocyte plasma membrane-associated protein	APMAP	-0.1186	0.5690
Hemoglobin subunit beta	HBB	-0.1069	0.7930
Biotinidase	BTD	-0.1022	0.6930
Heparin cofactor 2	SERPIND1	-0.1004	0.4860
Peroxiredoxin-1	PRDX1	-0.1003	0.8110
Complement factor D	CFD	-0.0983	0.8610
Vitamin K-dependent protein C	PROC	-0.0910	0.7900
Leucine-rich alpha-2-glycoprotein	LRG1	-0.0831	0.6810
Cartilage acidic protein 1	CRTAC1	-0.0807	0.9040
Beta-2-microglobulin	B2M	-0.0778	0.6810
Secretoglobin family 3A member 2	SCGB3A2	-0.0748	0.8730
Mannan-binding lectin serine protease 1	MASP1	-0.0665	0.7150
Insulin-like growth factor-binding protein complex acid labile subunit	IGFALS	-0.0610	0.9090
Apolipoprotein C-II	APOC2	-0.0405	0.8760
Ubiquitin carboxyl-terminal hydrolase 14	USP14	-0.0362	0.9380
Vinculin	VCL	-0.0322	0.9330
Ras-related protein Rab-14	RAB14	-0.0319	0.9090
Histone H2B type 1-K	H2BC12	-0.0281	0.9630
L-lactate dehydrogenase A chain	LDHA	-0.0271	0.9350
Thymosin beta-4	TMSB4X	-0.0213	0.9570
Alpha-fetoprotein	AFP	-0.0185	0.9650
Immunoglobulin heavy constant gamma 3	IGHG3	-0.0172	0.6890
Coagulation factor V	F5	-0.0065	0.9820
Caveolae-associated protein 2	CAVIN2	-0.0019	0.9980

Afamin	AFM	0.0078	0.9570
Proteasome subunit alpha type-1	PSMA1	0.0079	0.9870
Protein S100-A6	S100A6	0.0088	0.9820
Keratin. type I cytoskeletal 9	KRT9	0.0099	0.9850
Immunoglobulin lambda variable 8-61	IGLV8-61	0.0136	0.9560
Gelsolin	GSN	0.0167	0.5250
Apolipoprotein M	APOM	0.0182	0.9250
Vitamin D-binding protein	GC	0.0278	0.8830
Complement factor B	CFB	0.0287	0.8630
Alpha-1-acid glycoprotein 1	ORM1	0.0338	0.9330
Tubulin alpha-4A chain	TUBA4A	0.0354	0.7880
Immunoglobulin kappa light chain	IGK	0.0363	0.8260
Immunoglobulin heavy variable 4-28	IGHV4-28	0.0407	0.8690
Heat shock 70 kDa protein 1A	HSPA1A	0.0410	0.9350
Insulin-like growth factor-binding protein 6	IGFBP6	0.0431	0.8400
Immunoglobulin kappa constant	IGKC	0.0462	0.8400
Immunoglobulin heavy variable 2-26	IGHV2-26	0.0481	0.8490
Thyroxine-binding globulin	SERPINA7	0.0520	0.8550
Plexin domain-containing protein 2	PLXDC2	0.0541	0.8510
Fibulin-1	FBLN1	0.0542	0.7980
Carbonic anhydrase 1	CA1	0.0573	0.9250
Alpha-enolase	ENO1	0.0580	0.8570
Hemopexin	HPX	0.0605	0.8530
Immunoglobulin lambda variable 7-46	IGLV7-46	0.0638	0.8230
Transthyretin	TTR	0.0649	0.7610
Immunoglobulin kappa variable 2-30	IGKV2-30	0.0665	0.8630
Receptor-type tyrosine-protein phosphatase F	PTPRF	0.0681	0.7590
Cell division control protein 42 homolog	CDC42	0.0704	0.8910
Protein S100-A11	S100A11	0.0757	0.8130
Coagulation factor XI	F11	0.0764	0.8280
Vascular cell adhesion protein 1	VCAM1	0.0797	0.7560
CD5 antigen-like	CD5L	0.0808	0.8620
Vimentin	VIM	0.0848	0.9020

Cadherin-13	CDH13	0.0886	0.9380
Peroxiredoxin-6	PRDX6	0.0908	0.8820
Protein AMBP	AMBP	0.0929	0.6640
Inter-alpha-trypsin inhibitor heavy chain H2	ITIH2	0.0942	0.3440
Immunoglobulin heavy constant gamma 2	IGHG2	0.0959	0.6390
Transferrin receptor protein 1	TFRC	0.1087	0.6210
Glyceraldehyde-3-phosphate dehydrogenase	GAPDH	0.1107	0.7410
Hepatocyte growth factor activator	HGFAC	0.1109	0.6920
Angiotensinogen	AGT	0.1124	0.6300
Properdin	CFP	0.1219	0.8120
Complement component C7	C7	0.1259	0.6280
Complement factor H-related protein 2	CFHR2	0.1317	0.7800
Immunoglobulin lambda variable 1-36	IGLV1-36	0.1406	0.7870
N-acetylmuramoyl-L-alanine amidase	PGLYRP2	0.1437	0.5460
Immunoglobulin heavy variable 3-7	IGHV3-7	0.1470	0.3820
Beta-2-glycoprotein 1	APOH	0.1470	0.4810
Apolipoprotein E	APOE	0.1500	0.2100
Superoxide dismutase [Cu-Zn]	SOD1	0.1518	0.5130
Apolipoprotein C-I	APOC1	0.1528	0.6090
Immunoglobulin lambda-1 light chain	IGL1	0.1579	0.3020
Coagulation factor XIII B chain	F13B	0.1618	0.3310
Plasminogen	PLG	0.1640	0.2540
Carboxypeptidase B2	CPB2	0.1646	0.5620
Immunoglobulin lambda variable 2-8	IGLV2-8	0.1813	0.4920
Immunoglobulin lambda constant 2	IGLC2	0.1832	0.2130
Mannan-binding lectin serine protease 2	MASP2	0.1900	0.3620
Alpha-2-HS-glycoprotein	AHSG	0.2004	0.6590
Tetranectin	CLEC3B	0.2024	0.3860
Immunoglobulin lambda variable 9-49	IGLV9-49	0.2085	0.4160
Complement C4-A	C4A	0.2090	0.2200
Serum amyloid P-component	APCS	0.2099	0.6170
Phosphatidylinositol-glycan-specific phospholipase D	GPLD1	0.2114	0.7090
Complement C1s subcomponent	C1S	0.2123	0.1460

Immunoglobulin heavy variable 1-18	IGHV1-18	0.2130	0.5220
Probable non-functional immunoglobulin kappa variable 2D-24	IGKV2D-24	0.2180	0.4060
Collagen alpha-1(XVIII) chain	COL18A1	0.2210	0.5850
Immunoglobulin lambda variable 3-16	IGLV3-16	0.2219	0.4780
Inter-alpha-trypsin inhibitor heavy chain H4	ITIH4	0.2235	0.2080
Immunoglobulin lambda variable 2-23	IGLV2-23	0.2335	0.4250
Carboxypeptidase N subunit 2	CPN2	0.2341	0.6160
Vasodilator-stimulated phosphoprotein	VASP	0.2359	0.5960
Complement component C8 beta chain	C8B	0.2366	0.0673
Protein S100-A8	S100A8	0.2411	0.7020
Alpha-mannosidase 2	MAN2A1	0.2496	0.0900
Gamma-glutamyl hydrolase	GGH	0.2497	0.5310
Complement factor H-related protein 3	CFHR3	0.2506	0.4310
Complement component C9	C9	0.2515	0.6770
Immunoglobulin lambda constant 7	IGLC7	0.2542	0.5510
Integrin-linked protein kinase	ILK	0.2570	0.6230
Histone H4	H4C1	0.2673	0.6480
IgGFc-binding protein	FCGBP	0.2726	0.3260
Prothrombin	F2	0.2727	0.2800
CYFIP-related Rac1 interactor B	CYRIB	0.2864	0.5120
Probable non-functional immunoglobulin kappa variable 6D-41	IGKV6D-41	0.2883	0.3930
Desmoglein-2	DSG2	0.2886	0.1080
Low affinity immunoglobulin gamma Fc region receptor III-A	FCGR3A	0.2997	0.2200
Plastin-2	LCP1	0.3010	0.5080
Stress-induced-phosphoprotein 1	STIP1	0.3030	0.6230
Collectin-11	COLEC11	0.3047	0.3770
Inter-alpha-trypsin inhibitor heavy chain H1	ITIH1	0.3093	0.1660
Complement component C6	C6	0.3136	0.1090
Pigment epithelium-derived factor	SERPINF1	0.3163	0.1630
Ficolin-3	FCN3	0.3201	0.0880
Apolipoprotein(a)	LPA	0.3221	0.1990
Methanethiol oxidase	SELENBP1	0.3295	0.5030

Haptoglobin	HP	0.3366	0.2150
Receptor-type tyrosine-protein phosphatase S	PTPRS	0.3378	0.1850
Cathelicidin antimicrobial peptide	CAMP	0.3403	0.4430
GTP-binding nuclear protein Ran	RAN	0.3426	0.4470
Complement C1q subcomponent subunit B	C1QB	0.3484	0.2010
Myosin regulatory light chain 12B	MYL12B	0.3502	0.4650
Immunoglobulin lambda-like polypeptide 1	IGLL1	0.3511	0.4810
Peptidase inhibitor 16	PI16	0.3514	0.5460
Proteasome subunit alpha type-3	PSMA3	0.3514	0.5300
Endoplasmic reticulum chaperone BiP	HSPA5	0.3570	0.1110
Immunoglobulin lambda variable 6-57	IGLV6-57	0.3580	0.2530
Ras-related protein Rab-11B	RAB11B	0.3624	0.1210
Phosphoglycerate kinase 1	PGK1	0.3629	0.3480
Immunoglobulin heavy variable 3-15	IGHV3-15	0.3640	0.0580
Antithrombin-III	SERPINC1	0.3660	0.4340
Immunoglobulin heavy constant gamma 4	IGHG4	0.3680	0.3080
Neutrophil gelatinase-associated lipocalin	LCN2	0.3710	0.4390
Immunoglobulin heavy variable 5-51	IGHV5-51	0.3710	0.0660
Protein S100-A12	S100A12	0.3729	0.5810
Keratin. type II cytoskeletal 1	KRT1	0.3750	0.2560
Prolyl endopeptidase FAP	FAP	0.3765	0.2300
Exostosin-like 2	EXTL2	0.3846	0.2510
Lysozyme C	LYZ	0.4016	0.2390
Proprotein convertase subtilisin/kexin type 9	PCSK9	0.4036	0.1360
Serine/threonine-protein phosphatase 2A 65 kDa regulatory subunit A alpha isoform	PPP2R1A	0.4063	0.5850
Immunoglobulin kappa variable 3-11	IGKV3-11	0.4074	0.0870
Immunoglobulin heavy variable 3-74	IGHV3-74	0.4075	0.0640
Angiopoietin-related protein 3	ANGPTL3	0.4211	0.2280
Immunoglobulin J chain	JCHAIN	0.4211	0.0940
Immunoglobulin kappa variable 1D-13	IGKV1D-13	0.4242	0.2470
Sulfhydryl oxidase 1	QSOX1	0.4259	0.2210
Immunoglobulin kappa variable 1D-8	IGKV1D-8	0.4280	0.1560
Proteoglycan 4	PRG4	0.4288	0.1510

Immunoglobulin lambda variable 4-69	IGLV4-69	0.4301	0.0640
Complement C1r subcomponent	C1R	0.4372	0.3410
14-3-3 protein zeta/delta	YWHAZ	0.4388	0.0820
Complement factor H	CFH	0.4407	0.1050
Fermitin family homolog 3	FERMT3	0.4416	0.4270
Vitamin K-dependent protein S	PROS1	0.4445	0.3360
Vitronectin	VTN	0.4453	0.4260
Immunoglobulin lambda variable 1-47	IGLV1-47	0.4476	0.0720
Cholesteryl ester transfer protein	CETP	0.4483	0.2570
Immunoglobulin lambda variable 2-11	IGLV2-11	0.4510	0.1490
Complement component C8 alpha chain	C8A	0.4541	0.3940
Serum amyloid A-4 protein	SAA4	0.4630	0.1740
Immunoglobulin kappa variable 2-40	IGKV2-40	0.4631	0.0980
Latent-transforming growth factor beta-binding protein 1	LTBP1	0.4665	0.1060
Immunoglobulin heavy variable 1-3	IGHV1-3	0.4672	0.3530
Immunoglobulin kappa variable 2D-29	IGKV2D-29	0.4682	0.2550
Tenascin-X 5	TNXB	0.4697	0.0650
Nidogen-1	NID1	0.4702	0.1330
Immunoglobulin kappa variable 4-1	IGKV4-1	0.4725	0.2600
Apolipoprotein B-100	APOB	0.4753	0.1400
Collagen alpha-1(VI) chain	COL6A1	0.4774	0.3330
Immunoglobulin heavy variable 3-30	IGHV3-30	0.4779	0.2490
Protein S100-A4	S100A4	0.4917	0.2260
C4b-binding protein beta chain	C4BPB	0.4927	0.3040
Basement membrane-specific heparan sulfate proteoglycan core protein	HSPG2	0.5032	0.3720
Immunoglobulin delta heavy chain	IGD	0.5045	0.0641
Alpha-hemoglobin-stabilizing protein	AHSP	0.5067	0.3220
Procollagen C-endopeptidase enhancer 1	PCOLCE	0.5083	0.2080
Immunoglobulin heavy variable 4-34	IGHV4-34	0.5228	0.1010
Immunoglobulin heavy variable 2-70	IGHV2-70	0.5313	0.1200
Thrombospondin-4	THBS4	0.5317	0.0700
Lumican	LUM	0.5368	0.0850

Immunoglobulin heavy variable 3-23	IGHV3-23	0.5457	0.1320
Complement C1q subcomponent subunit C	C1QC	0.5468	0.1570
Dermcidin	DCD	0.5522	0.0950
Immunoglobulin heavy variable 3-49	IGHV3-49	0.5528	0.0660
Neutrophil defensin 1	DEFA1	0.5554	0.2340
Annexin A7	ANXA7	0.5555	0.2040
Immunoglobulin heavy variable 6-1	IGHV6-1	0.5598	0.1070
Actin-related protein 3	ACTR3	0.5651	0.2250
Extracellular matrix protein 1	ECM1	0.5661	0.2050
CD99 antigen	CD99	0.5663	0.3770
Immunoglobulin heavy variable 1-69	IGHV1-69	0.5666	0.0790
Transketolase	TKT	0.5677	0.2890
Pyruvate kinase PKM	PKM	0.5681	0.2350
6-phosphogluconate dehydrogenase. decarboxylating	PGD	0.5752	0.1410
NAD-capped RNA hydrolase NUDT12	NUDT12	0.5910	0.4560
Collagen alpha-1(I) chain	COL1A1	0.6311	0.1070
Haptoglobin-related protein	HPR	0.6512	0.2140
Phospholipid transfer protein	PLTP	0.6566	0.2230
Immunoglobulin alpha-2 heavy chain	IGA2	0.6653	0.2780
Immunoglobulin lambda variable 5-37	IGLV5-37	0.7094	0.0760
Importin subunit beta-1	KPNB1	0.7247	0.1790
Fibronectin	FN1	0.7596	0.0906
Hemoglobin subunit gamma-1	HBG1	0.8092	0.3110
Amyloid beta precursor like protein 1	APLP1	0.8157	0.0590
Talin-1	TLN1	0.9849	0.5780
Myeloperoxidase	MPO	0.9981	0.3140
Pregnancy zone protein	PZP	1.0662	0.4190
C4b-binding protein alpha chain	C4BPA	1.0666	0.1690
Lactotransferrin	LTF	1.0814	0.1230
Alpha-actinin-4	ACTN4	1.3922	0.0610

Table S5A. Enrichment analysis of proteins exclusively identified in cord-blood-derived exosomes from appropriate-for-gestational-age infants (AGA, N=20). Pathways are arranged according to its *p*-value (from top to bottom).

Category	Term	Count	P value
KEGG	Leukocyte transendothelial migration	4	6.2E-03
KEGG	TGF-beta signaling pathways	3	4.8E-02
KEGG	MAPK signaling pathways	4	7.3E-02
KEGG	Hippo signalling	3	9.3E-02
KEGG	Cell Cycle	3	9.4E-02
KEGG	Phagosome	3	9.5E-02
REACTOME_PATHWAY	Immune system	21	8.5E-06
REACTOME_PATHWAY	Neutrophil degranulation	9	3.0E-04
REACTOME_PATHWAY	Hemostasis	9	1.7E-03
REACTOME_PATHWAY	Cytokine signaling in immune system	10	1.7E-03
REACTOME_PATHWAY	Signaling by Rho GTPases	8	1.3E-02
REACTOME_PATHWAY	Metabolism of proteins	14	3.3E-02
REACTOME_PATHWAY	Signal transduction	15	7.6E-02
REACTOME_PATHWAY	Post-translational protein modification	10	8.5E-02

Table S5B. Enrichment analysis of proteins exclusively identified in cord-blood-derived exosomes from small-for-gestational-age infants (SGA, n=20). Pathways are arranged according to its *p*-value (from top to bottom).

Category	Term	Count	P value
KEGG	Proteasome	3	4.3E-03
KEGG	HIF-1 signaling pathway	3	2.2E-02
KEGG	AGE-RAGE signalling pathway in diabetic complications	3	1.9E-02
REACTOME_PATHWAY	Neutrophil degranulation	10	5.3E-06
REACTOME_PATHWAY	Innate immune system	11	5.2E-04
REACTOME_PATHWAY	Extracellular matrix organization	6	1.5E-03
REACTOME_PATHWAY	Cellular response to chemical stress	5	2.3E-03
REACTOME_PATHWAY	Metabolism of proteins	14	3.4E-03
REACTOME_PATHWAY	Nervous system development	7	4.6E-03
REACTOME_PATHWAY	Post-translation protein modification	11	6.7E-03
REACTOME_PATHWAY	RAF/MAPK cascade	5	6.8E-03
REACTOME_PATHWAY	Signal transduction	14	2.2E-02
REACTOME_PATHWAY	Hemostasis	6	3.1E-02
REACTOME_PATHWAY	Developmental biology	8	9.6E-02

Figure S2A. Receiver operating characteristic (ROC) curve of selected up-regulated proteins belonging to a network.

Only those with an area under the curve (AUC)>0.8 are depicted

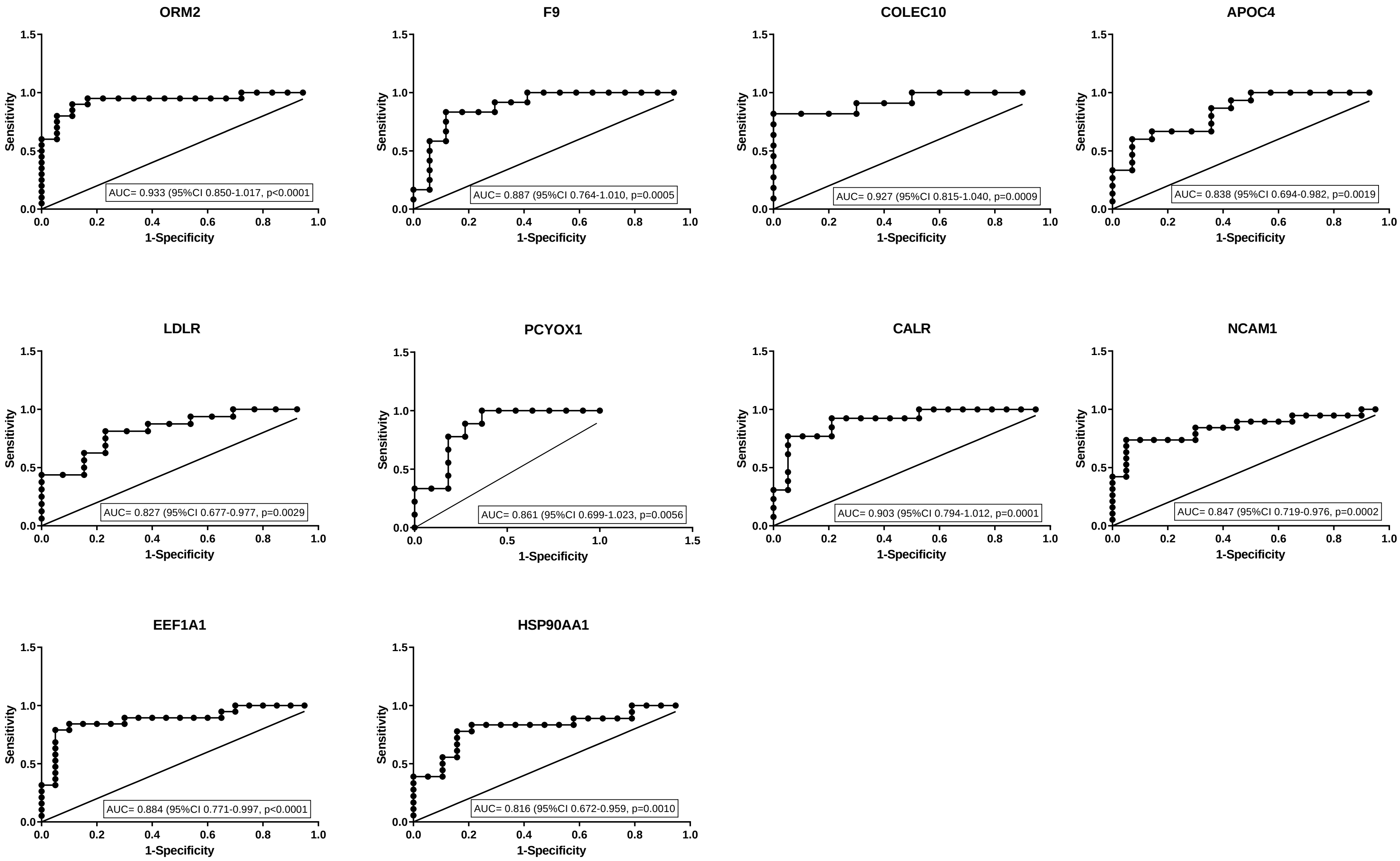

Figure S2B. Receiver operating characteristic (ROC) curve of selected down-regulated proteins belonging to a network.

Only those with an area under the curve (AUC)>0.8 are depicted

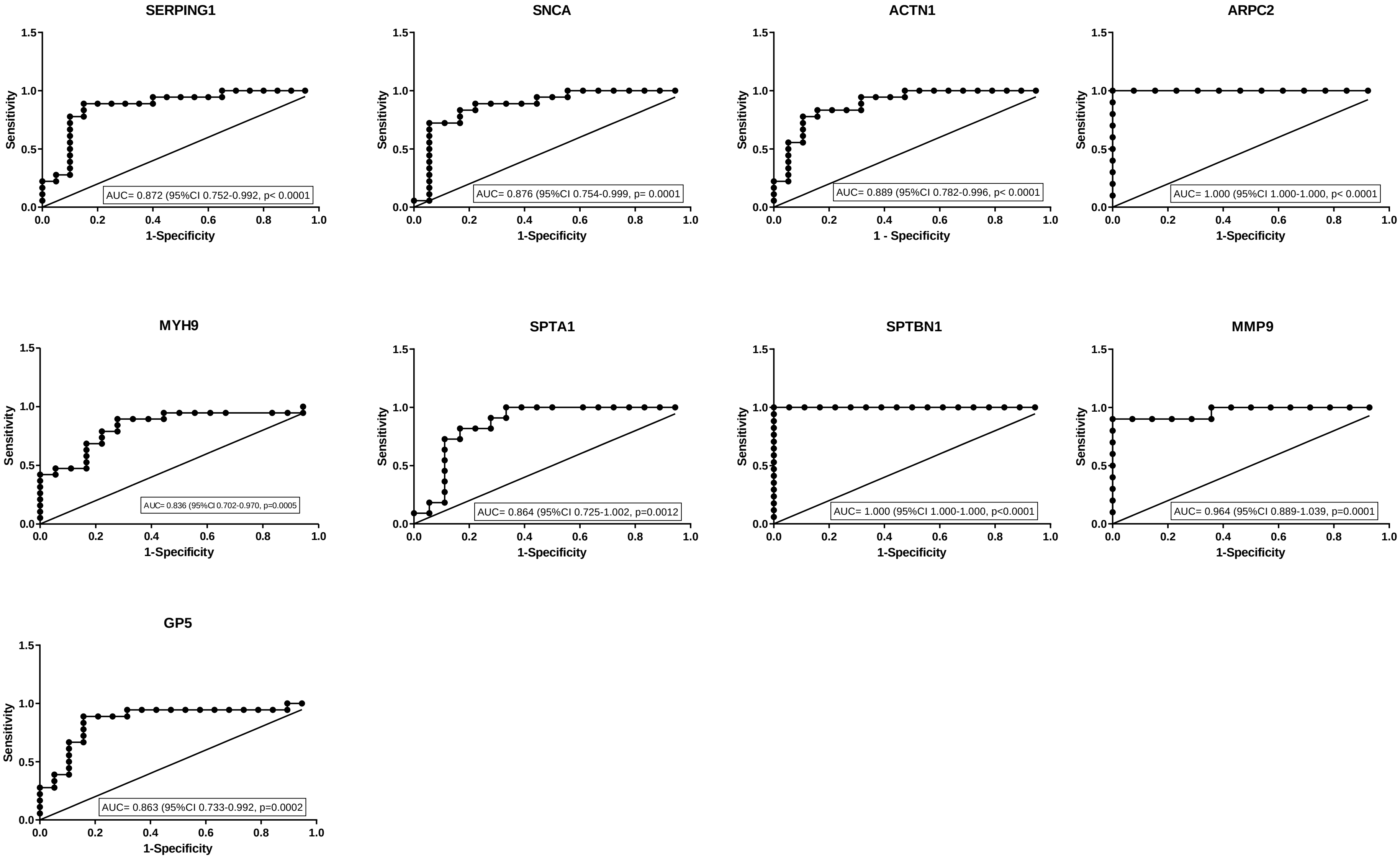

Figure S3. Correlation matrix of selected proteins with differential expression between appropriate-for-gestational-age (AGA, n=20) and small-for-gestational-age (SGA, n=20) subgroups and selected variables at birth

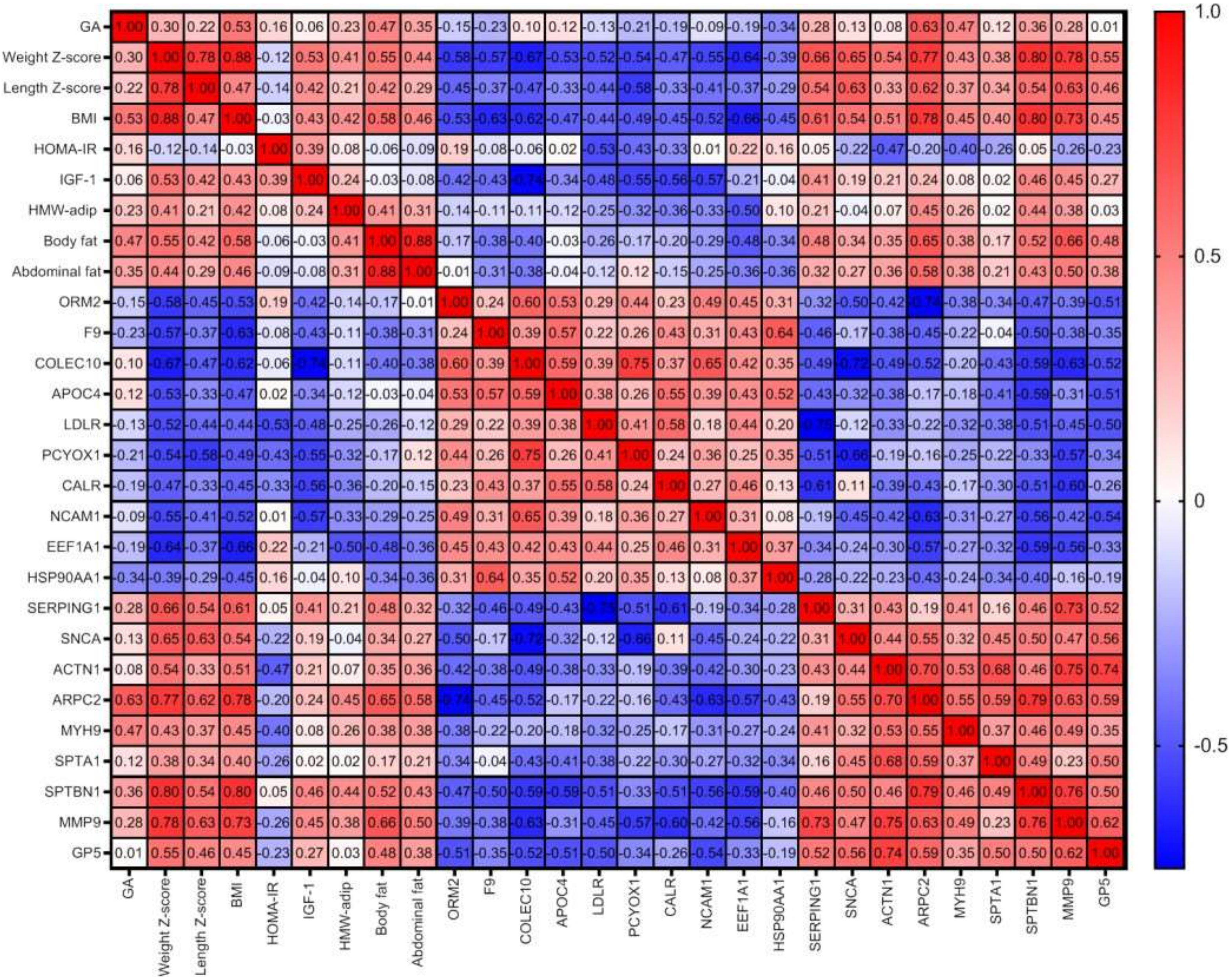

Figure S4. Correlation matrix of selected proteins with differential expression between appropriate-for-gestational-age (AGA, n=20) and small-for-gestational-age (SGA, n=20) subgroups and selected variables at age 2 years

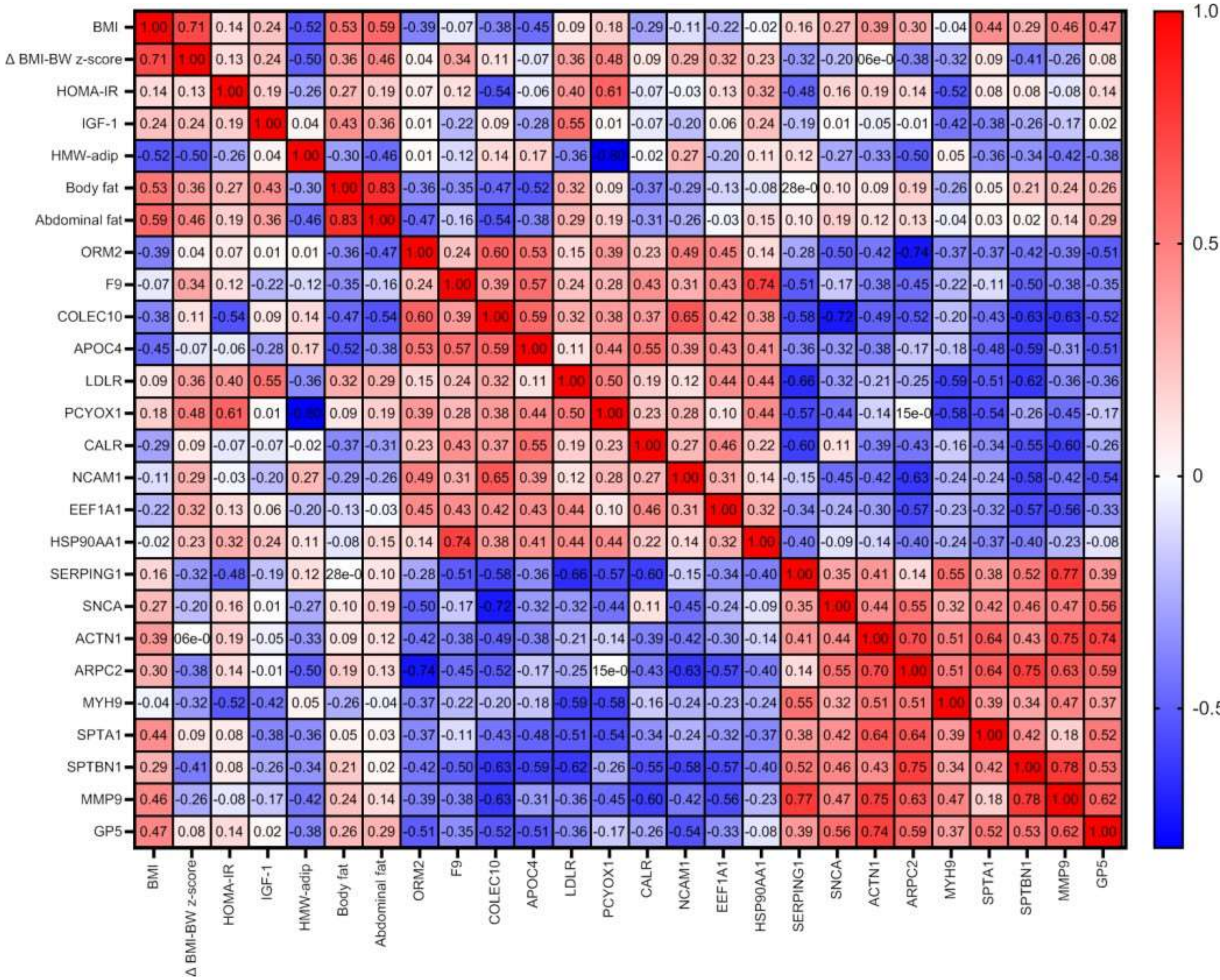

Figure S5. Correlation matrix of selected proteins with differential expression between appropriate-for-gestational-age (AGA, n=20) and small-for-gestational-age (SGA, n=20) subgroups and selected variables at age 7 years

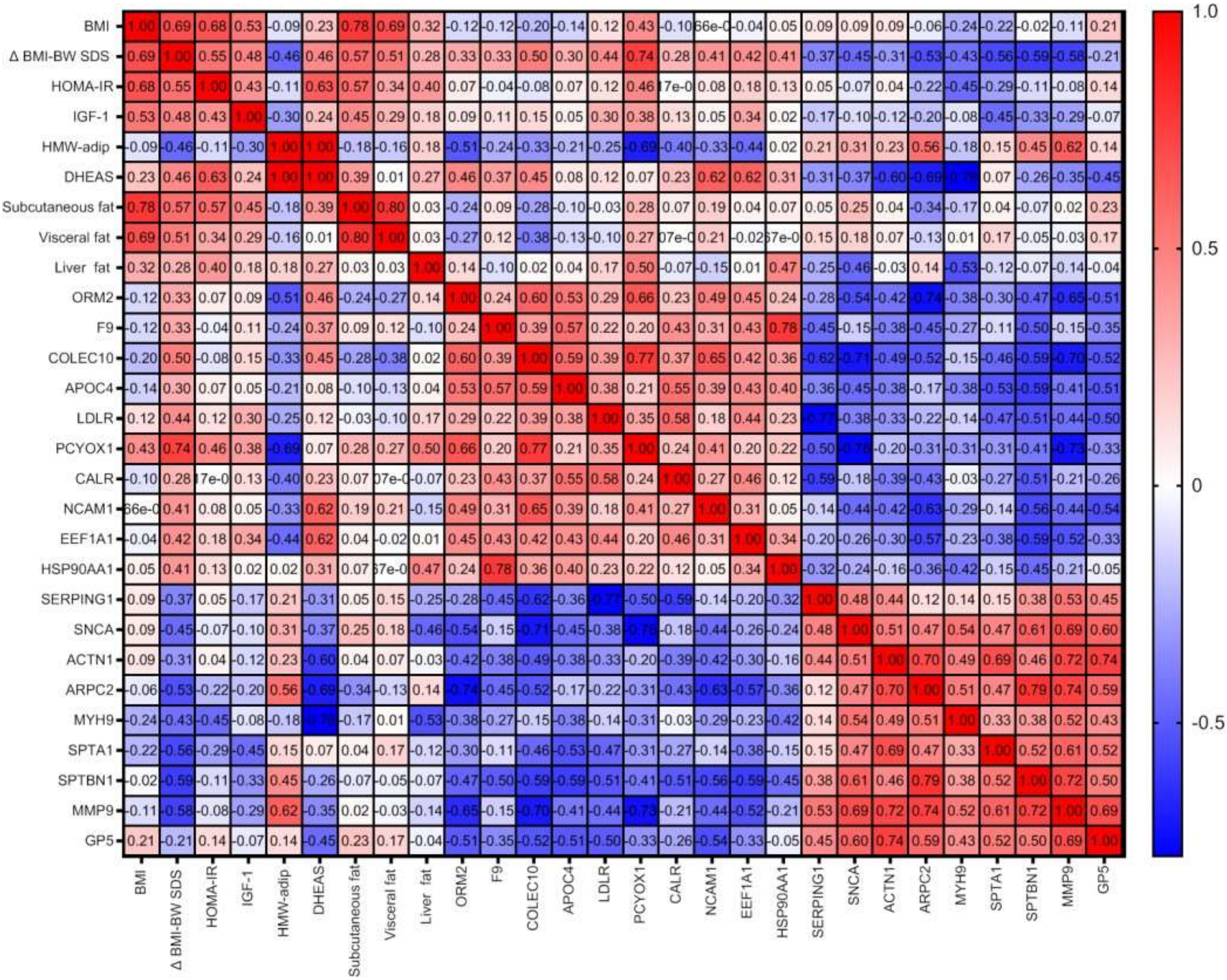

Table S6. Multivariate linear model of selected auxological, metabolic, body composition and proteomic parameters.

	[a]Fat mass at age 2 years		
	**Beta**	**Sig.**	R^2
**COLEC10**	0.811	0.015	0.601
	**[b]Abdominal fat mass at age 2 years**		
	**Beta**	**Sig.**	R^2
**CALR**	0.738	0.037	0.468
	**[c]Z-score change from BW to BMI at age 7 years**		
	**Beta**	**Sig.**	R^2
**SPTBN1**	-0.702	0.035	0.420
	**[d]HMW-adip at age 7 years**		
	**Beta**	**Sig.**	R^2
**MMP9**	-0.939	0.010	0.842

[a]Non-predictive variables: ORM2, F9, APOC4, LDLR, PCYOX1, CALR, NCAM, EEF1A1, HSP90AA1

[b]Non-predictive variables: ORM2, F9, COLEC10, APOC4, LDLR, PCYOX1, NCAM, EEF1A1, HSP90AA1

[c]Non-predictive variables: SERPING1, SNCA, ACTN1, ARPC2, MYH9, SPTA1, MMP9, GP5

[d]Non-predictive variables: SERPING1, SNCA, ACTN1, ARPC2, MYH9, SPTA1, SPTBN1, GP5

Figure S6. Consecutive recruitment of the AGA and SGA subpopulations

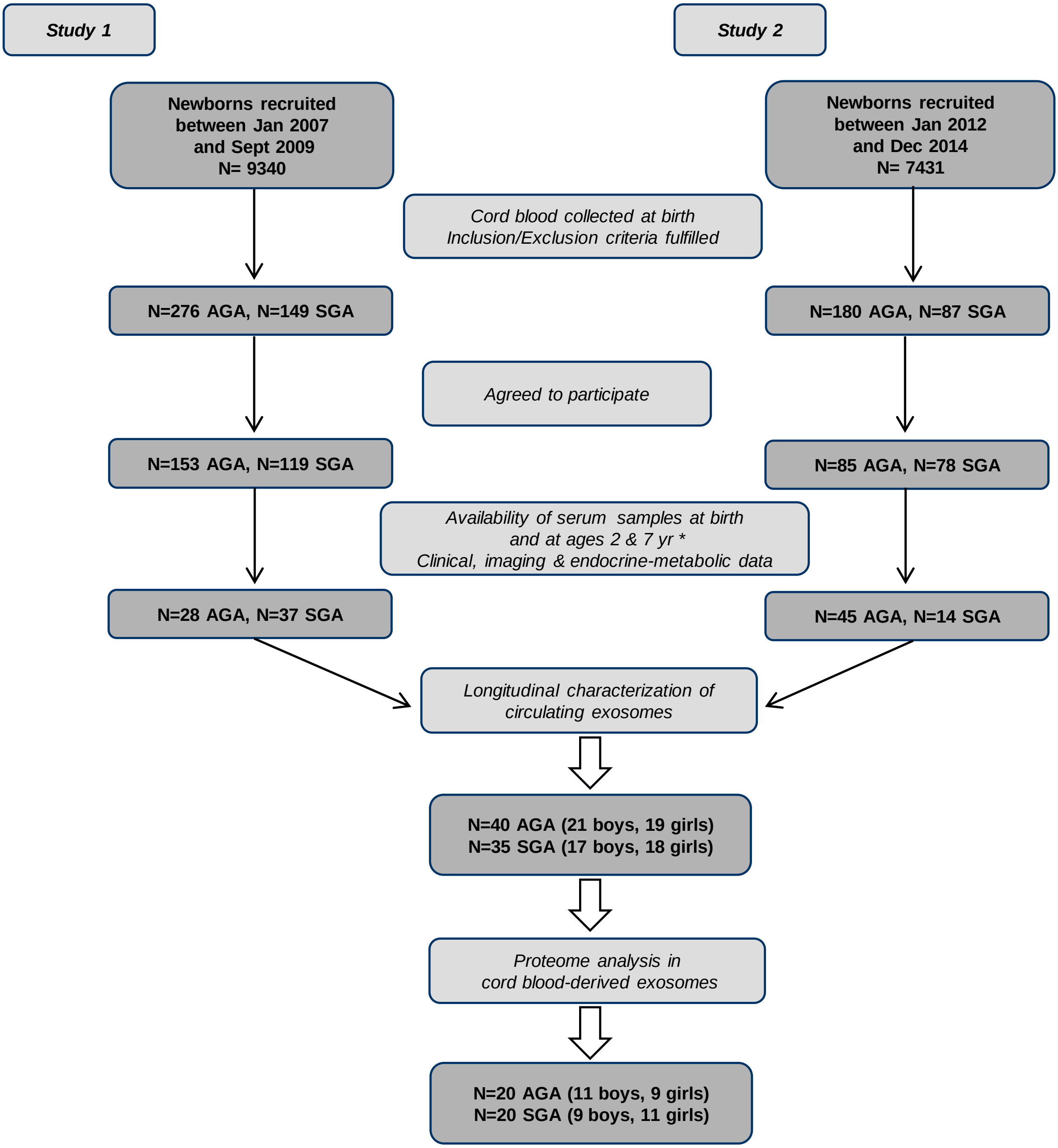

AGA: Appropiate-for-gestational-age
SGA: Small-for-gestational-age
*A minimum of 250 µl of serum is needed for exosome purification
